# Unveiling the Structural and Mechanical Diversity of SARS-CoV‑2 Variants Using Atomic Force Microscopy

**DOI:** 10.1021/acsami.6c03486

**Published:** 2026-06-01

**Authors:** Dominik Sziklai, Bálint Budavári, Bálint Kiss, Levente Herényi, Zoltán Kis, Bernadett Pályi, Miklós Kellermayer

**Affiliations:** † Department of Biophysics and Radiation Biology, 37637 Semmelweis University, Budapest, Pest county 1094, Hungary; ‡ HUN-REN-SE Biophysical Virology Research Group, 37637 Semmelweis University, Budapest, Pest county 1085, Hungary; § National Biosafety Laboratory, National Center for Public Health and Pharmacy, Budapest, Pest county 1097, Hungary

**Keywords:** coronavirus, nanomechanics, virion geometry, bending rigidity, membrane vesicle, structural biology, nanoscale biophysics

## Abstract

Understanding the structure and virion–host interaction of SARS-CoV-2 is crucial for elucidating the fundamental mechanisms of its assembly, stability, and transmission. These insights not only improve antiviral strategies but also contribute to a broader understanding of the nanoscale biological systems. Most studies of coronavirus focus primarily on viral genetics and on the structure and receptor affinity of the spike protein but overlook the broader mechanical and structural properties of the virion as a whole. Several studies have already suggested structural variability among coronavirus virions, and our work aims to provide more evidence of this matter. Here, we studied chemically fixed SARS-CoV-2 variants, focusing on wild-type, alpha, and delta variants. We used atomic force microscopy to acquire high-resolution topographic information. To estimate physically plausible virion envelope shapes, we utilized the Helfrich vesicle model as part of the analysis pipeline. We estimated viral geometry and adhesional compliance through reduced volume and its relationship with other geometrical parameters. We revealed consistent differences in apparent virion geometry across the three variants with alpha and delta displaying smaller fitted envelopes and lower reduced-volume estimates than wild-type under identical capture, fixation, and imaging conditions. Geometry-derived contact metrics also differed systematically among variants, consistent with differences in apparent deformation and compliance in this assay. Together, these descriptors establish a comparative framework for assessing the variant-dependent virion geometry and apparent deformation behavior.

## Graphical abstract

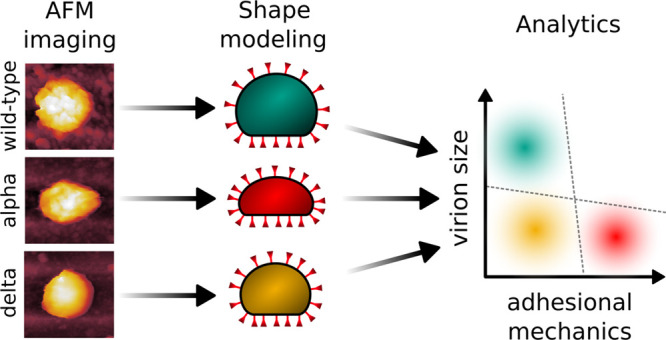

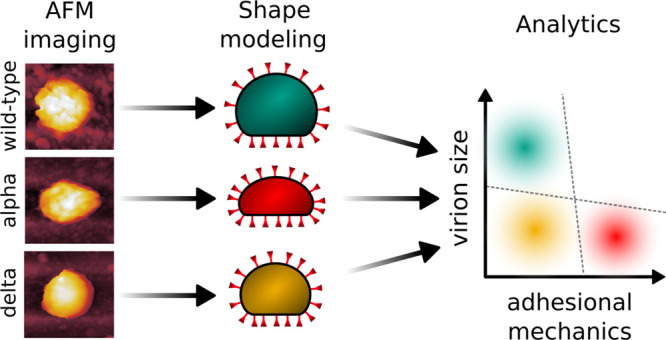

## Introduction

SARS-CoV-2 (severe acute respiratory syndrome coronavirus 2) is an enveloped, positive-sense, single-stranded RNA virus that primarily causes mild infections of the upper and/or lower respiratory tract.^1^ The severity of symptoms, disease progression, and prognosis are influenced by various factors.^2^ The recent pandemic has revealed that gaining a deeper understanding of this virus and its intricate properties is of great importance.

The coronavirus virion (Figure 1a) has three key roles: storing, protecting, and transporting the viral genome, mediating entry into the host cell, and escaping the host cell.^3–5^ The structure, size, and arrangement of the virion are physically shaped by the viral genome, the lipid envelope, and the structural proteins, which are the spike (S), envelope (E), membrane (M), and nucleocapsid (N) proteins.

**Figure 1:**
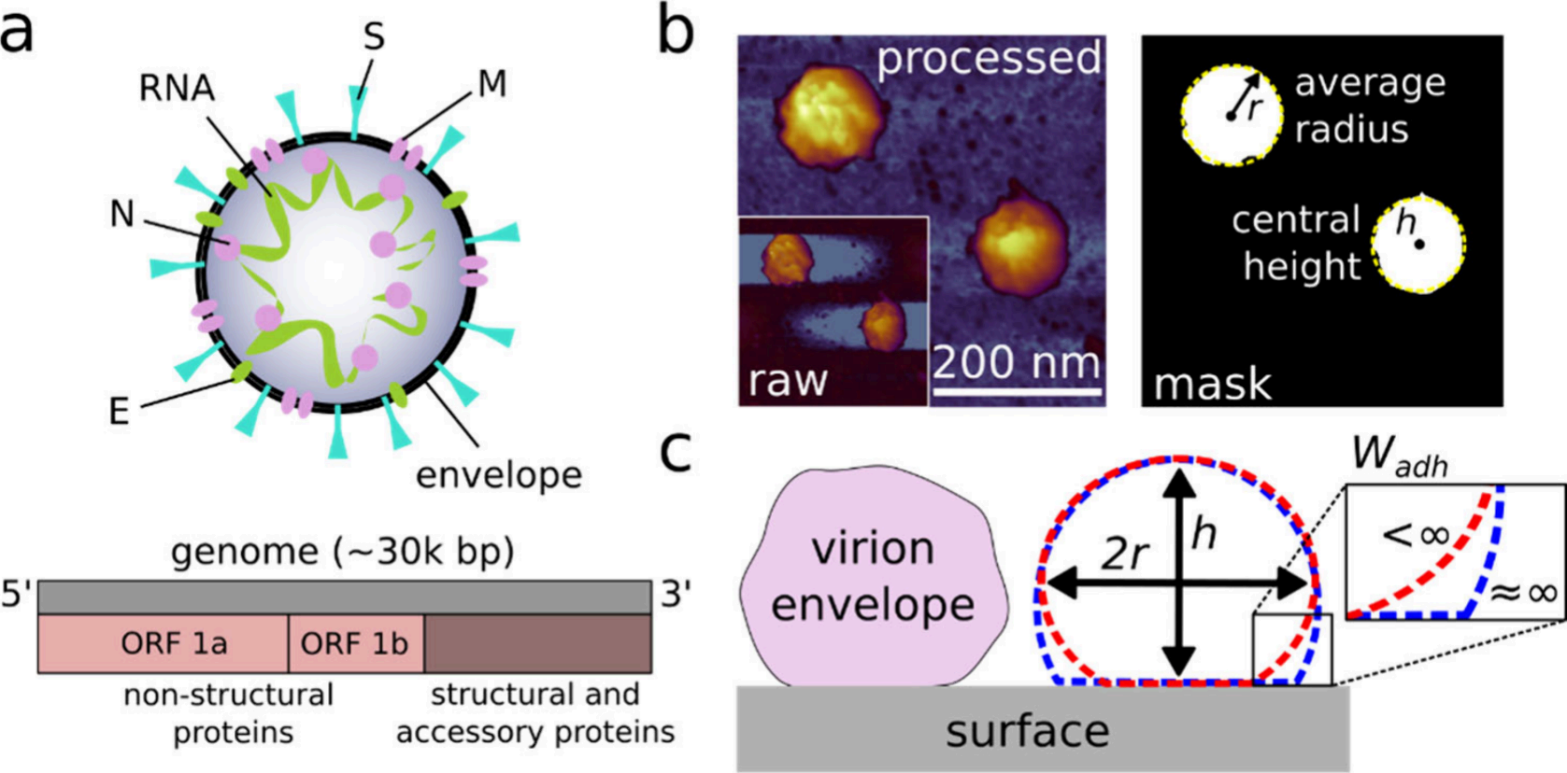
Structure, imaging, and modeling of coronavirus virions. (a) Schematic representation of a coronavirus virion and its components. At the bottom, a schematic gene layout is given of the coronavirus genome. (b) AFM images of coronavirus particles (raw and processed) and masked coronavirus particles to determine the average radius and central height for every particle. (c) Modeling the shape of a coronavirus virion, using the (blue, starting with a nonzero slope) Young-Dupré wetting and (red, starting with 0 slope) Helfrich vesicle models. The inset shows why the vesicle–surface contact is inappropriate in case of the Young-Dupré model since it assumes infinite adhesion potential (*W*_adh_; see Text S1), thus generating an invalid geometry.

Viruses evolve rapidly under constant selection pressure with high mutation rates.^6,7^ In SARS-CoV-2, this evolution resulted in novel variants having amino acid differences in their spike and other structural and nonstructural proteins, potentially altering their structure and interaction with the environment and host cells.^8^

SARS-CoV-2 research has focused mostly on the spike protein, a type-1 fusion protein essential for host recognition and membrane fusion.^9^ Mutations in the spike protein gene can lead to conformational alterations affecting the receptor-binding domain (RBD) and its resulting interaction with the host cell.^10^ This alteration directly affects the force profile of the entry process at the variant level, as was demonstrated with AFM in ref 11. However, in addition to spike proteins, the interplay of all structural components and environmental factors collectively and simultaneously impact virus–host interactions.^11–16^ This realization motivates us to compare different variants in terms of their geometries and apparent adhesional properties. These characteristics can directly affect practical applications^17–21^ in biotechnology or in general nanomedicine.

The internal medium of the coronavirus particle is assumed to be an aqueous environment resembling intracellular conditions.^3^ Imaging studies using cryo-EM, EM, and AFM show that the particle’s shape is nearly spherical with slight oval deviations, confirming its pleomorphic nature^22,23^ (Figure 1b). This geometric flexibility may facilitate infection by increasing the available contact surface area,^24–26^ and it may also influence translational and rotational diffusion.^27^

AFM images provide topographical data of coronavirus particles from which observables such as height and radius can be extracted (Figure 1b). As the surface is scanned by the tip, the recorded topography partially represents the envelope of the virus, the basis of which is a closed lipid bilayer vesicle.^12,28,29^

The virion diameter reported earlier ranges from 60 to 140 nm^23,30^ with variations stemming from several factors. Different imaging protocol steps, such as dehydration during sample preparation, can shrink particles, and fixatives like glutaraldehyde may change the apparent size.^31,32^ The complexity of virion assembly and the intracellular environmental changes may further contribute to the size variations,^33,34^ explaining the discrepancies in measured dimensions across studies.

Geometrical modeling of a vesicle attached to a surface can be effectively carried out by using the Young-Dupré wetting phenomenon.^14,35^ In this framework, the vesicle shape is approximated as a spherical cap (Figure 1c) by using the vesicle’s height and radius. Although this model provides a reasonable geometrical approximation, it is inaccurate and unphysical at the vesicle–surface contact.

To overcome this limitation, we employed the Helfrich vesicle model (also known as the spontaneous curvature model), a type of membrane energy model that can be used to describe vesicle shapes analytically. The model can also explain the shape of a vesicle attached to a surface (Figure 1c);^36,37^ for details, see Text S1.

Undoubtedly, the most important structures of the virion, the ones that make it infective, are the spike proteins. These spikes are constrained to the vesicle membrane and serve as partially flexible probes constantly in motion,^10,23^ allowing fusion with a host membrane. On topographical AFM images, spikes are difficult to distinguish;^23^ however, because they are crucial components of coronaviruses, we also included them in our analysis.

In this study, we used AFM topographical images to reveal the geometrical and adhesional compliance differences among early SARS-CoV-2 variants. *Wild-type*, *alpha*, and *delta* were selected because they represent temporally consecutive, epidemiologically important pre-*omicron* lineages with authentic isolate availability and well-documented differences in relative fitness, enabling a tractable comparison of virion-scale physical phenotypes across an early evolutionary trajectory. To produce realistic geometries, we implemented the Helfrich vesicle model to extract envelope shapes on which we put the spike protein layer as a shell to estimate geometrical features. Our geometrical analysis focused on the possible virus–host interaction surfaces and scales, gaining insights into how well each variant might be utilizing its available structural and functional components. Our results suggest a diverse and variable virus–host interaction geometry, also indicating different variant nanomechanics.

## Results and Discussion

### Topographical Features of SARS-CoV-2 Variants Are Highly Different

Representative AFM images of variants are shown in Figure 2a. AFM measurements revealed that the geometrical observables of SARS-CoV-2 variants vary considerably (Figure 2b–d, Table 1, Table S1, Figure S13).

**Figure 2:**
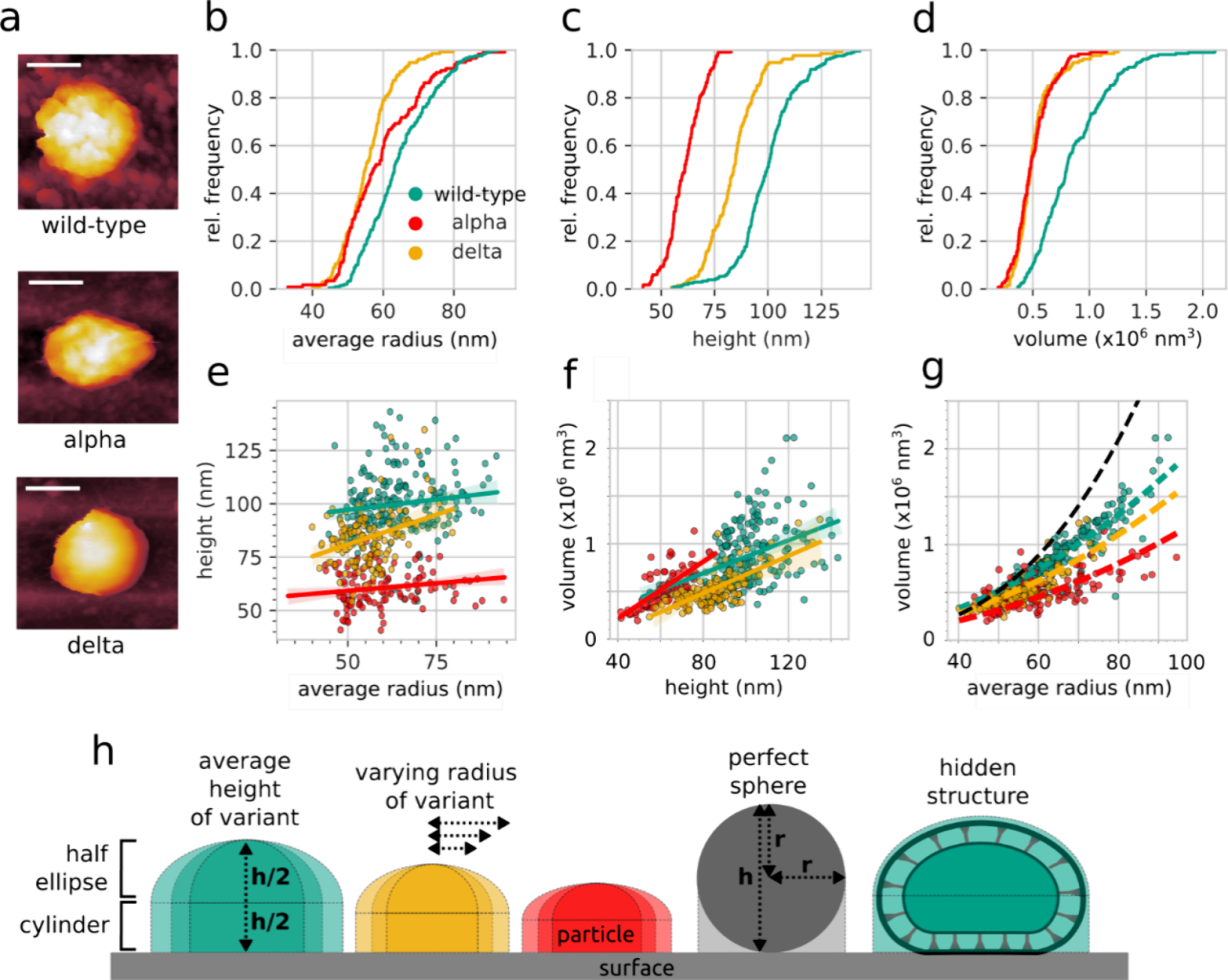
AFM measurements. (a) Representative AFM scans of variants (scale bar 50 nm). Empirical cumulative distribution functions of (b) radius, (c) height, and (d) volume. (e) Height vs radius, (f) volume vs height, and (g) volume vs radius relations for the different variants (solid lines are linear regressions). Colored dotted curves in (g) present the expected volume as a function of radius for 3D shapes presented in (h). The black dotted curve shows the expected volume for a perfect sphere as a function of radius. (h) Schematics of the approximate geometry of variants expected for perfect nonconvoluted AFM images. These shapes are scaled according to the measurement values and are constructed by a half ellipse and a cylinder. These constructions explain the apparent coronavirus geometries. A reference shape is also shown for a perfect sphere (black). It should be noted how the real structure of the virion is buried under the topography (last shape). *n* = 230 *wild-type*, 130 *delta*, and 124 *alpha*.

**Table 1:** AFM Measurement Results for Each Variant^a^

variant	mean of average radii (nm)	mean height (nm)	volume (×10^6^ nm^3^)	circularity
*wild-type*	65 ± 10	100 ± 15	0.85 ± 0.30	0.85 ± 0.06
*alpha*	59 ± 8	61 ± 12	0.46 ± 0.13	0.87 ± 0.06
*delta*	56 ± 7	83 ± 11	0.48 ± 0.12	0.86 ± 0.05

a Data are shown as mean ± SD. The definition of circularity is given in the Methods: Image Processing and Data Collection section. Statistical evaluation is in Figure S13 and Table S1. *n* = 230 *wild-type*, 130 *delta*, and 124 *alpha.*

The *alpha* variant had the lowest and the *wild-type* had the greatest topographical height, while the height of *delta* particles was intermediate. The volume distribution of the *wild-type* coronavirus had the greatest standard deviation (SD) compared to the other two variants, and it had an overall greater mean than the other ones. The *wild-type* had the greatest radius. The nearly identical circularity values indicate similar lateral footprint roundness across variants, whereas the more pronounced differences in height and volume point to differences in vertical flattening/inflation state under the same assay conditions. We analyzed the correlations between measured variables to gain insights and quantify their possible relations (Tables 2 and S4).

**Table 2:** Correlation Coefficients (*r*) Derived from Measurement Data^a^

variant	radius vs height	height vs volume	radius vs volume
*wild-type*	0.12	0.42	0.90
*alpha*	0.09	0.70	0.65
*delta*	0.31	0.71	0.82

a Statistical evaluation is shown in Table S4. *n* = 230 *wild-type*, 130 *delta*, and 124 *alpha*.

While Figure 2e and Table 2 show weak correlations between the mean radius and the height, we found pronounced correlations between height and volume and even higher correlations for mean radius and volume, and notably, these correlations are slightly different for each variant (Figure 2f,g). The schematics in Figure 2h show the suggested geometries that explain these correlations. Hypothetically, variance in variant sizes should primarily lead to variance in their volumes. To model the AFM-measured volumes of surface-adhered virions, we can use the measured average radius and central height (Figure 1b). Using these two variables, topographical volumes can be estimated with an object: its upper half is a half-ellipse, and the lower half is a cylinder (Figure 2h), respecting the topographical nature of AFM images. When this simple geometric model was used with the average height of each variant and the radii were varied, these geometries replicate the observed volumes almost perfectly (Figure 2g). For comparison, the dashed black line in Figure 2g indicates what the expected volumes should be as a function of the radius for a perfectly spherical particle.

The range of the measured quantities is comparable to published parameters of coronaviruses but with significant differences among the variants (Figure S13 and Tables S1 and S4). Circularities of the variants show no significant difference, indicating that the equilibrium shape is circular (oblate in 3D) with only slight perturbations, which may be attributed to pleomorphic behavior.

Overall, our measurements are explained by the simple model geometry, and we confirm the consistent differences of variant sizes and shapes, as illustrated in Figure 2h. *Wild-type* is the largest and has a shape that is closest to that of a perfect sphere. *Delta* and *alpha* are smaller, and *alpha* exhibits a more flattened shape. These measurements can be further improved, and a more accurate estimation of the viral envelope can be achieved, though it is challenging as the envelope is buried inside the topographical AFM scans (Figure 2h). Our apparent-shape results should be read alongside prior AFM studies showing that SARS-CoV-2 virions are unusually compliant at the single-particle level and that morphology depends strongly on sample handling, surface chemistry, and imaging regime.^23,38^ Recent work has combined nanoindentation with simulation to infer the whole-virion mechanical response,^39^ and recent reviews place these measurements within the broader AFM virology toolkit.^40^

### Model Fitting Reveals Further Differences Between Variants

Variant envelope geometries were modeled by fitting the aforementioned Helfrich vesicle model on the measurement data (see Introduction and Text S1). To fit the model properly onto the envelope part of the virion, it is necessary to discard the spikes from the fit. AFM topographical data represent a height value for each pixel on the image. We know that the measured height of a virion is composed of different parts of the virus, as presented in Figure 3a,d: the top spike layer, the middle envelope layer, and the bottom spike layer. The diverse conformation and position of spike proteins^14,23^ and the spike-receptor overlapping on the bottom complicate the determination of exact height contributions of the internal layers. To stay consistent in our study, we always determined the height of a virion at the center point of the segmented mask, as presented in Figure 1b and Figure 3b.

**Figure 3:**
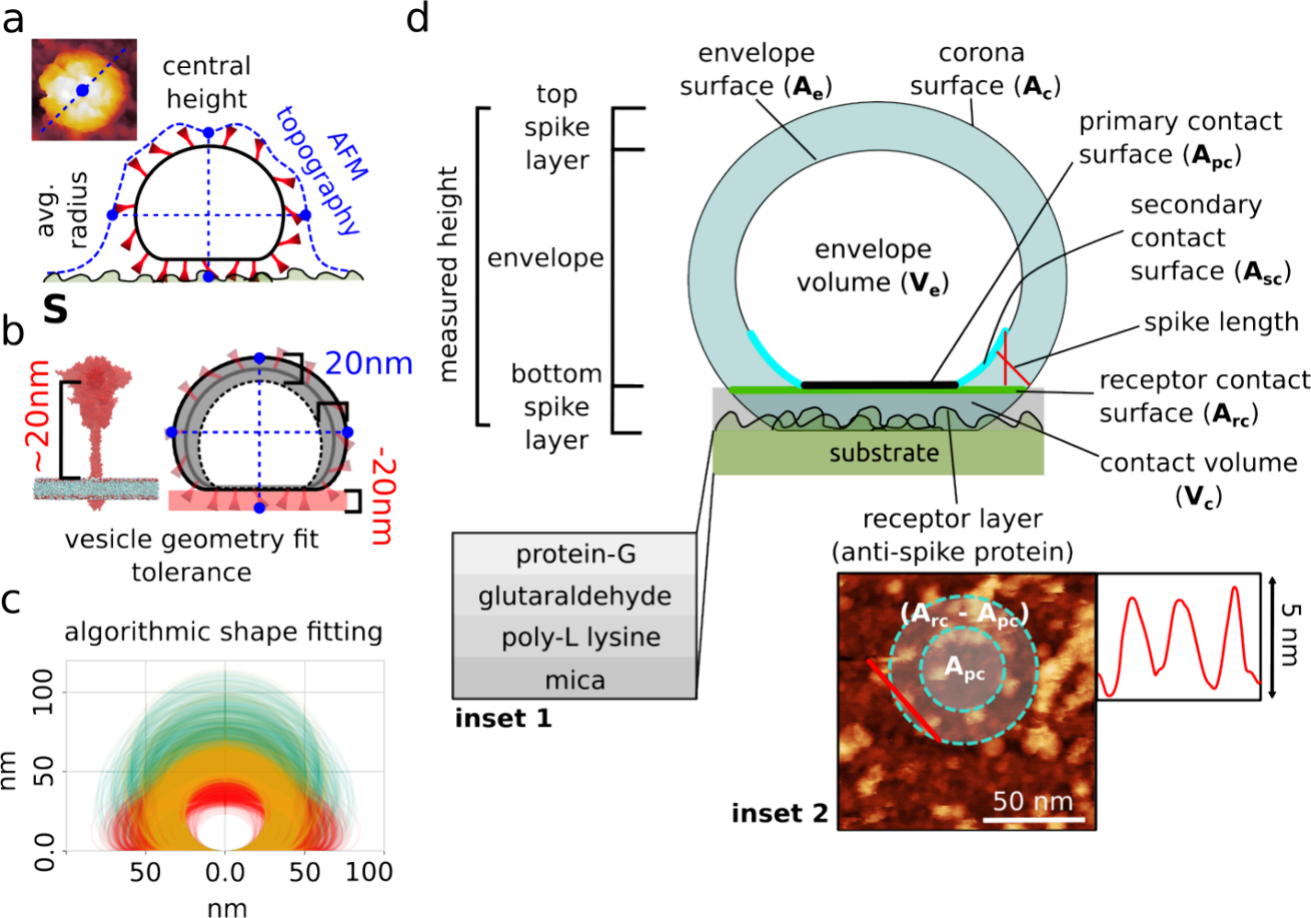
Overview of the analysis pipeline and schematics of analyzed geometries. (a) Schematic interpretation of a topographical AFM scan, depicting the uncertain contributions of the spike proteins to vesicle height and radius. (b) The uncertainties are handled by first subtracting the contribution of the bottom spike layer and then fitting the vesicle model on the envelope using the 20 nm tolerance handling the top and radial spike variability. (c) Depiction of the final envelope shape ensemble. (d) Visualization of the definitions of the geometric features. The spike length helps to determine *A*_sc_ and *A*_rc_, since these are bounded by the positions for which a full-length spike protein can still interact with the host (see also Figures S10 and S11). (Inset 1) The layers of the substrate surface in our experiments. (Inset 2) A representative AFM image of the surface on which coronaviruses were fixed. The red plot on the right shows the topography along the red line in the AFM image. The encircled areas present the *A*_pc_ and *A*_rc_ for a wild-type coronavirus virion.

The overview of the fitting process can be seen on Figure 3a–c, and exact details, considerations, accuracy, and sensitivity analysis are discussed in Texts S1–S3 and Figures S1–S7. We can approximate the full length of a spike protein outside the viral vesicle to be ∼20 nm^14,23,41^ (Figure 3b). The maximum height error (due to the top and bottom spike layers) is thus ∼2 × 20 = 40 nm. The bottom spike layer (spike-receptor overlap), due to the relatively extensive flat surface, most likely consists of ∼10 spike proteins (as we later present it, Figure 4a), and many of these spikes are probably captured by the surface-attached antibodies. Considering that multiple antibody-captured uniform spikes are contributing to this bottom layer, its height contribution should be stable and its uncertainty should be relatively small; this allows us to account for the bottom spike layer error by simply subtracting 20 nm from the total measured height (Figure 3a,b).

**Figure 4:**
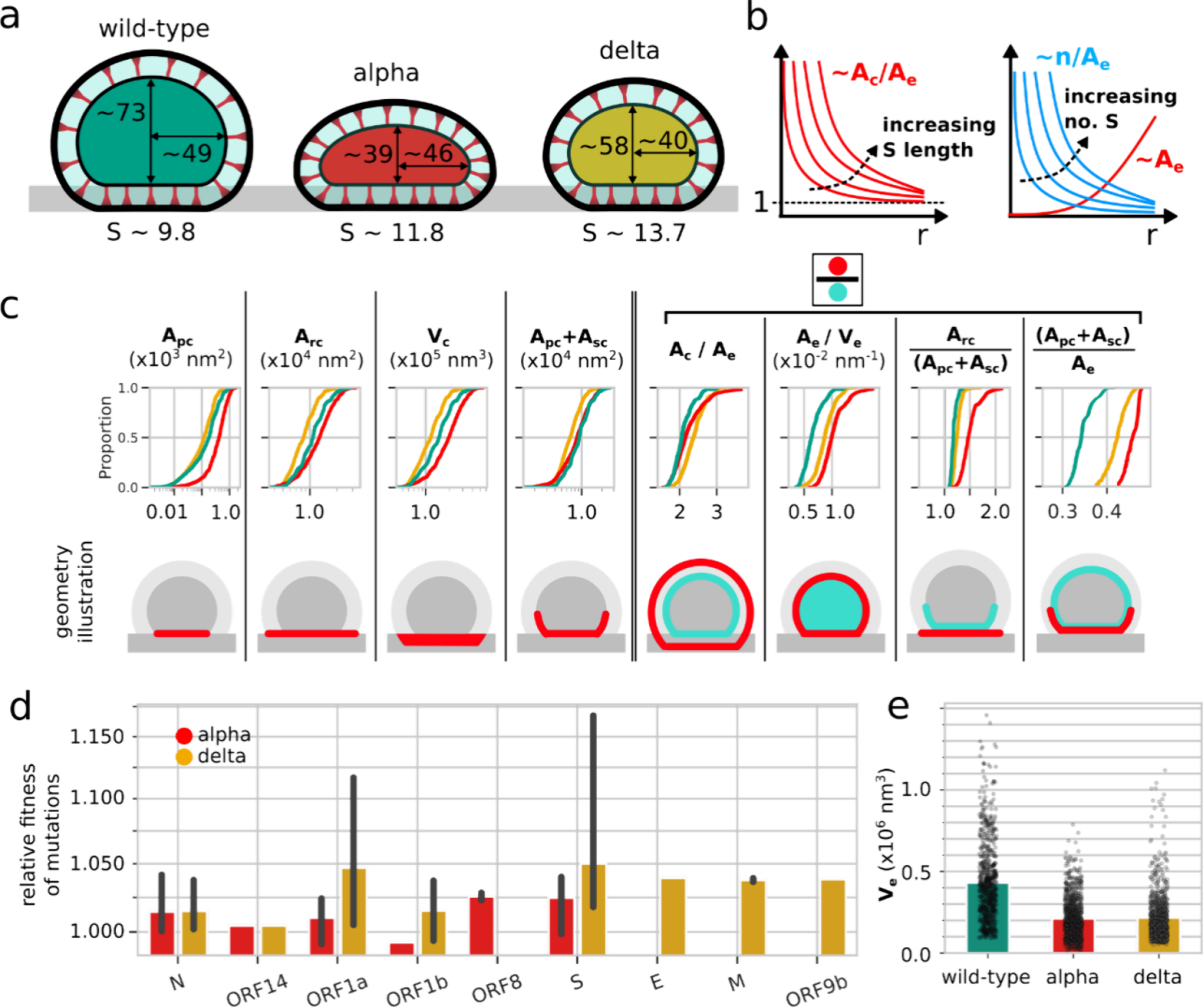
Geometrical comparison of the SARS-CoV-2 variant envelopes based on the Helfrich model. (a) Comparable depiction of the modeled virion shapes (height and radius in nm). Below each variant, the estimated number of spikes in the contact area (*A*_pc_ + *A*_sc_) is shown. (b) *A*_c_/*A*_e_ and *n*/*A*_e_ as a function of radius (*r*), where *n* is the number of S proteins in the envelope. A family of curves is shown on both plots for the different S protein lengths and different number of S proteins. (c) The distribution functions for the defined geometrical traits. Each geometry is illustrated below the graphs. For ratios, red-colored quantities are divided by blue-colored ones. Geometric definitions are detailed in Figure 3d. (d) The estimated mean relative fitness values per gene for the alpha and delta variants with standard deviations. These fitness values are relative to the wild-type. (e) Estimated envelope volumes of variants. *n* = 3228 *wild-type*, 2327 *delta*, and 2520 *alpha*.

Estimating the top spike layer height contribution was more complicated. To properly acquire the vesicle height, we measure the center point of the particle, where the vesicle height is supposedly maximal. However, since this is a single point, the interfering spike conformations can vary a lot, adding significant noise from measurement to measurement. It is even possible that in some measurements we do not have any spikes at this central point (Figure 3a,b). Since the maximum error for the top spike layer is 20 nm and the minimum is 0 nm (no spike present), we accept fitting results that are a maximum of 20 nm below the measured height (Figure 3b).

The same 20 nm error tolerance is applied for the measured average radius values (Figure 3b). This is justified as a high conformational variation is also expected for radial spikes, along with slight asymmetries. This tolerance scheme allows us to account for conformational uncertainty and generate physically constrained distributions of plausible envelope geometries for each variant rather than single-point estimates. Envelope fitting was performed for all available virions using each particle’s measured height–radius pair. Because the applied height and radius tolerances admitted multiple, geometrically plausible solutions per virion, each particle yielded an ensemble of admissible envelope shapes rather than a single fit (Figure 3c and Figures S2, S3, and S6), resulting in larger shape-level sample sizes (*n* = 3228 wild-type, 2327 delta, and 2520 alpha). These fitted shapes are not independent observations, as multiple solutions originate from the same virion; accordingly, they are interpreted primarily as descriptive ensembles of plausible geometries under the assay constraints, while the independent experimental units remain the virions themselves.

Table 3 shows the resulting envelope volumes (*V*_e_), envelope surface areas (*A*_e_), and envelope surface per volume (*A*_e_/*V*_e_) ratios. The external validity of our modeled geometrical properties is underlined by the fact that our estimated *A*_e_ values for the variants (Table 3) are almost identical with EM-based estimated envelope surface area values published in ref 34: ∼20,000 nm^2^ for *alpha*, ∼20,000 nm^2^ for *delta* and ∼24,000 nm^2^ for *wild-type*. This also verifies our assumption and algorithmic handling of the lower, upper, and radial spike layer structure, leading to successful identification of the envelope shapes. We observed no prominent within-variant clustering reflected in the analyzed data, so variant groups are assumed to be homogeneous.

**Table 3:** Geometry of the Fitted Models^a^

variant	*V*_e_ (×10^6^ nm^3^)	*A*_e_ (×10^5^ nm^2^)	*A*_e_/*V*_e_ (×10^–2^ nm^–1^)
*wild-type*	0.43 ± 0.21	0.27 ± 0.09	∼6
*alpha*	0.21 ± 0.12	0.20 ± 0.08	∼10
*delta*	0.22 ± 0.14	0.18 ± 0.07	∼8

a Envelope volume is *V*_e_; envelope surface area is *A*_e_. Data are shown as mean ± SD. Statistical evaluation is in Table S2 and Table S3. *n* = 3228 *wild-type*, 2327 *delta*, and 2520 *alpha*.

After fitting the vesicle model onto the virion envelopes, we also added a 20 nm-thick corona layer onto them (Figure 3d, Figure S1, and Text S3). On the virion, spikes can be bent, compressed, or sterically hindered and, therefore, often extend less than 20 nm. Using the 20 nm spike length nonetheless offers a simple, uniform proxy that highlights and compares spike-related geometric features at their maximal values with a known envelope shape. This standardized reconstruction enables a direct, like-for-like comparison of virion geometries across variants. We can identify and quantify these geometric features using the fitted shapes. These features are visualized in Figure 3d. The fitted mean envelope heights and radii for each variant are shown in Figure 4a.

Geometrical features are compared in Figure 4c. The first four blocks in Figure 4c measure the absolute quantities of the modeled features (Figure S14 and Table S2): the primary and secondary contact surfaces (*A*_pc_ and *A*_sc_) represent the limiting surface area on the envelope with which the virion can interact with the host through the spike proteins; these spikes reach to the bottom of the viral envelope (Figure S11). The receptor contact surface (*A*_rc_) represents the same concept but from the perspective of the host (so it can be useful for receptor contact calculations if we know the receptor density on the host cell surface). *A*_sc_ and *A*_rc_ are bounded by the points where the virion’s spike proteins can still reach the host (see Figure 3d) in a geometrical sense. This is a fair estimate, as spikes can bend almost 90° in specific scenarios utilizing their hinges.^42^ A visual guide for *A*_rc_ and *A*_pc_ is presented in Figure 3d, inset 2 for the *wild-type* coronavirus on AFM images, and further explanation and comparison are provided in Figures S10 and S11. Since there is a potential overlap between the corona layer and host surface proteins, and this is a 3-dimensional region, it is reasonable to also represent it as a contact volume (*V*_c_) for variant comparison (Figure 3d). By using the spike protein surface density estimations of *wild-type*: 1.0; *alpha*: 1.3; *delta*: 1.9 S per 1000 nm^2,^ ^34^ and assuming a uniform spike distribution, we can estimate the number of spike proteins available for surface attachment in our sample (Figure 4a) using the (*A*_pc_ + *A*_sc_ *)* surface. The estimated ∼10 spikes in the contact region refer to spikes that are geometrically available within the contact-accessible area (*A*_pc_ + *A*_sc_) under an assumed ∼20 nm corona layer and an approximately uniform spike distribution. It is therefore an upper-bound accessibility estimate, not a count of simultaneously engaged bonds. Using the whole envelope area (*A*_e_), the number of spikes on the virion is around 27, 25, and 33 for *wild*, *alpha* and *delta*, respectively. Notably, despite having the smallest total spike count, with its higher adhesional compliance, *alpha* can still line up more spikes in the contact region compared to the *wild-type* coronavirus.

Besides the absolute quantities, it is more informative to introduce ratios between specific geometrical traits and compare the variants along these axes. These normalized values can better capture the differences on a similar scale. The last four blocks of Figure 4c (and Figure S15, Table S3) compare the variants according to defined ratios.

The density of spike proteins is proportional to 1/*A*_e_, meaning that a lower *A*_e_ might be preferable. A higher *A*_e_ (thus lower spike density) can be compensated for by increasing the number of spikes (Figure 4b, right). The corona surface per envelope surface ratio (*A*_c_/*A*_e_) quantifies how effectively spikes extend the envelope surface (*A*_e_), creating a relatively larger surface for potential contacts. This relation is altered by changing the length of spikes (Figure 4b, left).

The envelope surface per volume ratio (*A*_e_/*V*_e_) represents how large the interacting surface is compared to the viral cargo, representing an overall measure of how well the virus utilizes available resources for infection. It is also called the specific surface area, which is an important quantity in the study of inorganic materials and nanoparticles.^25,43^ The ratio of receptor contact surface per primary + secondary contact surfaces (*A*_rc_/(*A*_pc_ + *A*_sc_)) expresses how the contacting virus and host surfaces relate to each other. As this ratio increases, the relative accessible host surface per virion increases. Finally, the ratio of primary + secondary contact surfaces per vesicle contact surface (*A*_pc_ + *A*_sc_)/*A*_e_ reveals what proportion of its total surface a variant can utilize for infection when adhering to a host. Figure 4e shows the fitted envelope volumes calculated for each variant. It can be instantly seen that the envelope volumes of the *alpha* and *delta* variants are very close to each other, while the envelope volume of the *wild-type* is well separated from both.

Thus, the fitted vesicle model, together with the corona layer, provided interesting insights into the geometrical features for each variant. Variants are definitely smaller in size than the *wild-type* coronavirus, this has already been suggested by ref 34. Our defined geometrical parameters and ratios emphasize the structural optimization of the virus from the perspective of virus–host interaction. Overall, the *alpha* and *delta* variants seem to dominate our defined geometrical features (e.g., specific surface area or contact surface relative to the virion surface) over the *wild-type*, suggesting that these shape parameters can be important in optimizing and enhancing infectivity. According to our measurements and estimations, the *alpha* variant has the most deflated envelope. It must be emphasized that these small absolute and relative geometrical differences are amplified by the huge and exponential viral load, that is, the millions of virions produced in the host organisms, making it possible that these small differences matter significantly on the large scale.

### Adhesional Differences Suggest Variant-Dependent Deformation and Compliance

Under sufficient adhesional force, a soft virion will partially deform (flatten), and a dynamically changing contact zone is established (Figure 5a), increasing the virion’s contact area (effectively raising its area-to-volume ratio^14^). Virion adhesion is a multicomponent and multiscale phenomenon, and the attachment relies on a combination of specific and nonspecific interactions mediated by a host of nanomechanical parameters (Figure 5b). Specific spike and nonspecific envelope interactions alter the contact potential *W* (adhesion energy per unit area),^14,24^ while membrane bending rigidity (κ) and in-plane membrane tension (σ) change resistance to deformation.^44^ Internal pressure *P* (volume constrain) and the spontaneous curvature (*C*_0_) due to the possible leaflet asymmetry of the envelope and internal scaffolding^12^ also affect virion shapes at contact. Empirically, AFM confirms that coronavirus particles compress by ∼20–50% of their diameter upon binding to substrates, with greater deformation occurring when the virus’s envelope directly adheres to the surface and slightly less, when spikes primarily mediate the attachment.^14^

**Figure 5:**
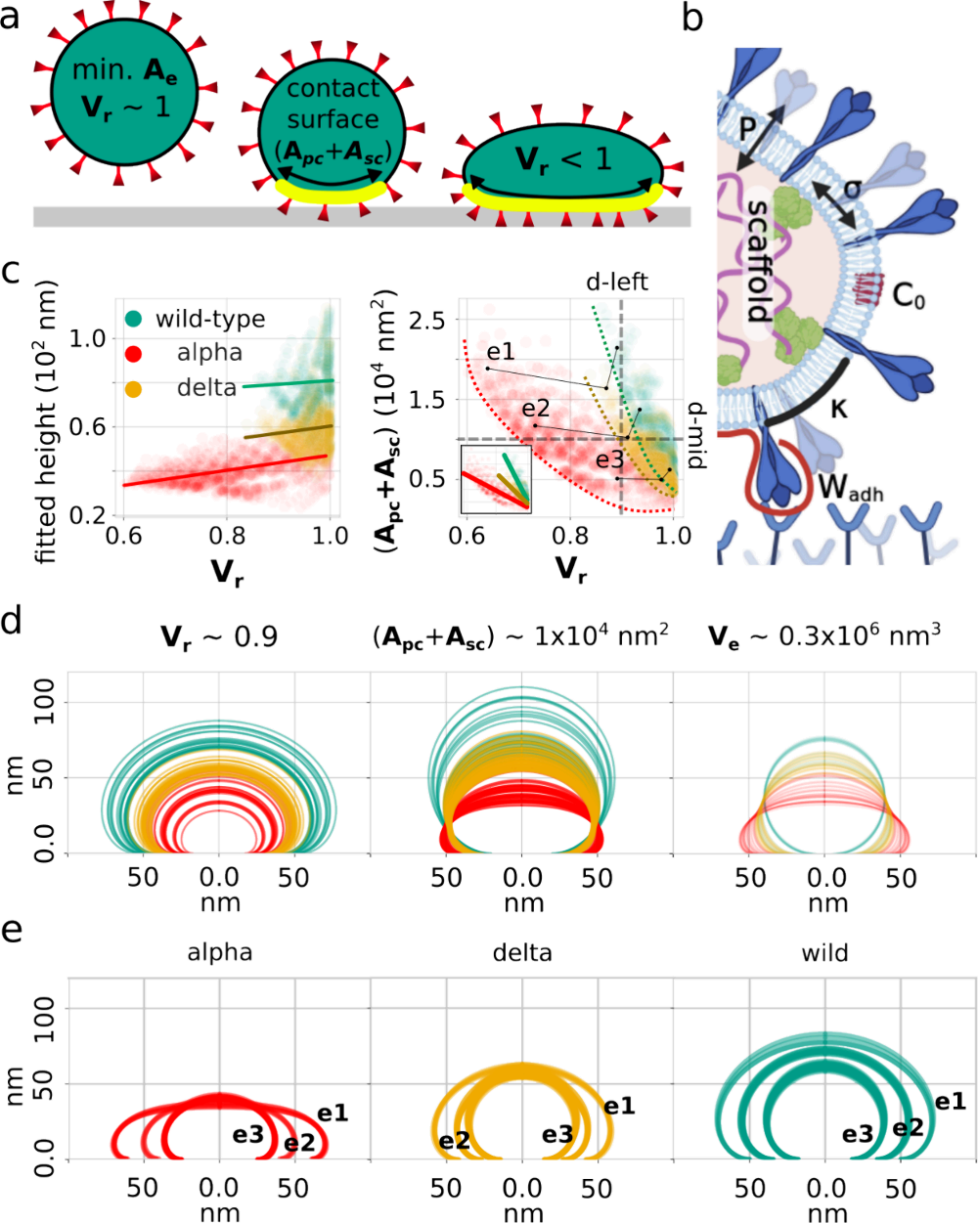
Differences in the adhesion profiles of variants. (a) Schematic representation of the virion adhesion event. The almost perfectly spherical virion approaches the surface. Once it comes into contact, either directly through the envelope or indirectly through spike interactions, the envelope starts deforming, as the contact surface dynamically forms until the energy balances. (b) A schematic collection of the relevant biophysical components that determine variant adhesion (see the text for their details). (c) (left) *V*_r_ vs fitted height and (right) *V*_r_ vs contact area (*A*_pc_ + *A*_sc_). The inset shows the fitted linear regression lines. The dotted lines present the implied minimal *V*_r_ boundary at which a given variant can reach that contact area. (d) Shape ensembles for several fixed properties. The fixed contact area (*A*_pc_ + *A*_sc_) and *V*_r_ shapes are extracted along the vertical and horizontal dashed lines on (c, right). (e) Representative envelope shapes sampled from (c, right) at the corresponding points (e1, e2, e3) for each variant.

The estimated shapes carry a relatively large structural uncertainty due to the presence of spike proteins (20 nm tolerance), but the analysis of the derived shape ensembles (Figure 3c) still reveals interesting statistical tendencies and clear insights into the virion envelopes. One can define the reduced volume (*V*_r_) as a dimensionless quantity expressing how closely a 3D object resembles a perfect sphere. Regarding our modeled envelope shapes, the smaller the *V*_r_, the more flattened the envelope is. Its maximum value is 1. The exact definition of *V*_r_ can be found in the Supporting Information (Text S4 and Equation S6). The different flatness values of variants is clearly indicated by the *V*_r_ estimations in our data (Table 4, Table S2, and Figure S14). *V*_r_ is therefore informative for within-assay comparison but should not be assumed to represent the native solution-state morphology independently of surface effects. Analyzing the estimated reduced volumes with related contact geometry reveals different adhesional behaviors and compliances across variants (Figure 5c).

**Table 4:** Linear Regression Slopes of the Analyzed Shapes^a^

variant	*V*_r_	*V*_r_ vs (*A*_pc_ + *A*_sc_) (10^4^ nm^2^)	*V*_r_ vs fitted height (nm)
*wild*	0.96 ± 0.02	–12.21 ± 0.21	3.55 ± 0.59
*alpha*	0.81 ± 0.09	–3.45 ± 0.82	2.45 ± 0.48
*delta*	0.94 ± 0.03	–7.03 ± 0.18	5.05 ± 0.47

a Data are shown as mean ± SD for *V*_r_ and SE for the slopes. Correlations are in Table S4. For *V*_r_, *n* = 3228 *wild-type*, 2327 *delta*, and 2520 *alpha*. For linear regressions, *n* = 230 *wild-type*, 130 *delta*, 124 *alpha*. For inferential comparisons of fitted shape parameters, each virion was treated as an independent experimental unit. For each parameter, all fitted shapes belonging to a given virion were summarized by their mean, and statistical comparisons were then performed on these virion-level mean values. Accordingly, the sample size for hypothesis testing was the number of virions, not the number of fitted shapes.

As expected for a vesicle-like structure on the surface, there is a slight increase in height as *V*_r_ increases (Figure 5c, left, corresponding slopes in Table 4). This observation supports the internal consistency of the fits and shows that the modeled shapes behave as expected for oblate envelopes, and we also clearly see the different height clusters of the three variants.

Figure 5d, left illustrates the envelope shapes at fixed *V*_r_ values. When envelope shapes are compared at a fixed *V*_r_, which standardizes the inflation state by controlling the ratio of surface area to enclosed volume, the resulting profiles reflect differences in absolute size (i.e., total area and volume) but not in overall geometry. In this representation, we observe that *wild-type* virions are the largest, followed by *delta*, while *alpha* is the smallest, even though all share nearly identical shape contours. Biophysically, this points toward biogenetic or structural differences across variants that manifest as variations in particle size and envelope extent, even when deformation behavior is matched. The *wild-type*’s larger size at the same *V*_r_ could imply a higher surface area reservoir, greater internal content, or a different particle maturation. In contrast, the *alpha* variant’s smaller envelope may reflect a more compact particle morphology, tighter envelope scaffolding, reduced internal load, or excess area. These distinctions provide further indication that variant physical state and envelope structure differ, even when apparent deformation compliance (via *V*_r_) is held constant.

The *V*_r_ vs contact area (*A*_pc_ + *A*_sc_) relationship can be seen in Figure 5c, right, and the corresponding slopes are in the inset and in Table 4. All three relationships are negative, so as *V*_r_ increases, contact surface decreases; this is again what is expected of oblate-shaped envelopes of approximately the same size. The up- and down-shifts of the lines are caused by the absolute size differences of the envelopes. The indicative limiting boundaries drawn in Figure 5c, right (dotted lines) present the minimum *V*_r_ (maximum flattening) the given variant can achieve to generate the given contact area. Adhesional compliance differences can also be interpreted as a (right–left) shift and skew of these limits. We emphasize, however, that *V*_r_ vs contact area (*A*_pc_ + *A*_sc_) slopes are used as comparative, geometry-derived descriptors of how fitted virion shapes populate contact-area states under nearly identical capture and imaging conditions. They should not be interpreted as direct measurements of bending rigidity, membrane tension, or the whole-virion elastic modulus.

Spreading characteristics can be quantified using the regression lines. In Figure 5c, right inset, the steepest absolute slope is produced by the *wild-type* coronavirus, reflecting its minimal spreading (stays close to *V*_r_ ∼ 1) and the contact area increase is primarily due to the increasing size of the virion particle, also indicated in Figure 5d, center. However, this behavior makes its relative quantities (Figure 4c) lag behind, indicating a less optimized structure. In contrast, for *alpha*, we see the smallest absolute slope, and the greatest *V*_r_ range, while *delta* is in-between the two.

Isovolumetric comparison of virions at fixed *V*_e_ (Figure 5d, right) reveals systematic differences in contact area between variants: *alpha* consistently exhibits the largest adhesion footprint, followed by *delta*, while the *wild-type* strain forms the smallest contact zones. This trend suggests that, within the present assay, the *alpha* variant shows the greatest apparent deformation under adhesion, consistent with more extensive flattening at the contact interface. In contrast, the *wild-type* virions show a lower apparent flattening under the present capture and fixation conditions. Since volume is held constant across all estimations in this analysis, the observed differences arise either from changes in effective membrane area (e.g., protein rearrangement) or from nanomechanical differences such as bending rigidity, tension, or envelope-scaffold coupling that govern how readily a particle spreads on the substrate.

In Figure 5e, we provide shape samples for comparison taken from Figure 5c, right, at the indicated positions. These characteristic shapes give visual intuition of the different sizes and possible envelope dynamics of the variants. How such adhesional behavior can be exactly broken down to the multitude of analytical components (Figure 5b) that participate in the phenomena is not possible without further assumptions.

The utilized antibody was primarily specific to the *wild-type* (see Methods, Substrate Surface Preparation), and this could raise the possibility that the *wild-type* virion was tethered by the spikes, while *alpha* and *delta* virions interacted with the surface directly through the envelope, as suggested before,^14^ thus leading to a flattened appearance. This cannot explain, however, why *delta* variant stays very inflated, close to the *wild-type*. Moreover, since our geometrical estimations (*A*_e_) are very close to the literature values for all variants,^34^ this supports the claim that the bottom spike layer (∼20 nm, Figure 3a,d) is present for all three variants. However, this does not prove identical spike-antibody capture affinity or identical tethering geometry. Another important aspect is the evolutionary changing of spike chargedness of variants.^45^ Charge accumulation of spike RBDs not only alter specific interactions but also affect the overall adhesion dynamics. Literature indicates that the formal charge of the trimeric spike head domain is +3, +6, and +18 for *wild-type*, *alpha*, and *delta*, respectively.^46^ This puts *delta* further from the other two variants, but as we see on our images, the shape of *delta* is closer to the *wild-type* than to *alpha*, making the electric effect less likely in this scenario. This is reinforced by the fact that our substrate surface (Figure 3d) is expected to exhibit a net neutral to slightly negative surface charge, as the positive poly-l-lysine,^47^ protein G (pI ∼ 4.5 ^48^) and IgG antibodies possess a weakly anionic character.^49^

Overall, the common assay conditions support comparison of the variants under similar experimental constraints with an estimated average spike protein density at the contact interface of approximately ∼10 spikes for all variants (Figure 4a). Nevertheless, variant-dependent differences in antibody engagement, nonspecific adhesion, or tethering geometry cannot be excluded. With these assumptions, our observations imply that the viral envelope’s nanomechanics and embedded proteins and their interaction and functional variability lead to the different apparent compliance of variants, allowing us to use reduced volume vs contact area slopes (Figure 5c, right, Table 4) as a rough all-in proxy for these effects.

The differences in the reduced volume vs contact area slopes show that *wild-type* and *alpha* are very far from each other in this sense, while *delta* is in between. We recall that the difference of estimated volumes (Table 3 and Figure 4e) for *alpha* vs *delta* variants is on the order of 10^4^ nm^3^, and the *wild* vs *alpha* and *delta* difference is an order of magnitude greater, 10^5^ nm^3^. Thus, the compliance and size tendencies move in different directions among the variants, indicating that these virion traits might be the result of different and possibly independent biological processes. The literature indicates that membrane nanomechanics is essential throughout the infection, affecting the dynamics of membrane pore formation and liquid flow profile.^50,51^ Certain antiviral chemicals lead to the stiffening of the virion membrane.^16^ A too compliant envelope, however, could even disrupt membrane fusion,^50^ so virion evolution presumably aims for a structural optimum to maximize biological fitness. In this context, the apparent differences in adhesional compliance observed under our experimental conditions may reflect subtle variant-dependent differences in envelope mechanics, although this interpretation remains indirect and requires further functional validation.

### Genotype–Phenotype Interplay

Genotypic variation is the primary driver of phenotypic differences.^7^ Point mutations in viral structural proteins can alter a virus’s mechanical and geometrical characteristics.^10,15,16^ However, data on such traits remain scarce for SARS-CoV-2 variants.^10^ To address this gap, we compiled relative fitness data to infer how structural differences might be encoded in the coronavirus genome. Figure 4d shows the relative fitness of key mutations in the *alpha* and *delta* variants based on data from ref 52. Both variants exhibit higher fitness than the *wild-type* strain. *Alpha* carries no significant mutations in the E, M, or ORF9b genes, whereas *delta* lacks mutations in ORF8 but harbors high-fitness substitutions in ORF1a, ORF1b, and S. Thus, *alpha*’s known mutations confer smaller fitness advantages than *delta*’s. Notably, the *omicron* variant (not shown) emerged with over 30 spike mutations and swiftly outcompeted *delta* globally.^34^

The fitness data suggest that *delta*’s competitive edge came from high-fitness mutations in its spike and ORF1a/1b (nsp) genes. Furthermore, *delta* harbors mutations in all four structural protein genes (S, E, M, N), whereas *alpha* has notable changes only in S and N. These structural proteins are key to viral assembly, stability, immune evasion, maturation, and release,^7,33^ and altering their arrangement can influence the flexibility of the viral membrane.^16,53^ No single mutation pattern clearly explains the smaller virion size or more deflated (flattened) shape observed in *alpha* particles. Indeed, recent cryo-electron tomography (cryo-ET) studies find that virion morphology remains largely spherical and unchanged across variants.^34^ For example, *omicron* BA.1 virions average ∼75 nm in diameter (BA.2 ∼ 82 nm), comparable to the ∼80 nm size of early SARS-CoV-2.^54^ If *alpha*’s slightly flatter appearance is due to a structural mutation, it likely lies in N or S. The nucleocapsid (N) protein is a strong candidate given its role in forming the RNP complex.^55^ Yao et al. described two RNP packing geometries (hexagonal and tetrahedral), where the tetrahedral form was more prevalent in elliptical virions, suggesting a link between RNP arrangement and particle shape.^55^

Although *alpha* and *delta* differ from *wild-type* by only a limited number of spike substitutions per monomer, mechanical consequences need not scale linearly with mutation count if substitutions affect interprotomer interfaces, conformational equilibria, or force-bearing pathways. At the same time, whole-virion compliance is a collective phenotype that also depends on the M/E/N organization, RNP packing, lipid composition, and assembly conditions.

Ultimately, some of these mutational effects may contribute to the higher viral loads observed in variants compared to the original strain.^56^ Rapid replication and maturation leading to high viral titers might correlate with the production of slightly smaller virions.^57,58^ Host factors such as age, sex, and immune status also modulate viral load^56^ and could influence virion production and shedding. Meanwhile, differences in the envelope lipid composition^16^ may affect variant stability or morphology, further complicating the search for a single genetic cause of these phenotypic differences. We propose that interplay between genetic changes and external factors drives the geometrical, nanomechanical, and adhesional diversity of SARS-CoV-2 variants. These observations suggest that variant evolution is not merely rewriting nucleotide sequences but also actively tuning the virion architecture and mechanics to enhance transmission. Establishing genotype-biophysics links will provide a testable predictive tool for future variant surveillance.

### Summary and Implications

To summarize our modeling and estimation workflow and its advantage, we first have to emphasize that topographical AFM imaging carries several inherent limitations, one of which is the projective nature of the images, which only allows us to scan the top of the sample. By using prior knowledge of the scanned objects shape (virions in this case), we can improve the accuracy of the information carried by our AFM images. Thus, to acquire more realistic shapes of coronavirus variants, we utilized Helfrich’s vesicle model to numerically estimate envelope shapes. Figure 6a,b shows how utilizing the model improves the interpretation of the measurement results; as shown in (a), the calculated reduced volumes (*V*_r_, Table 4 note, Equation S6) using the original measurements are indistinguishable and invalid (>1). However, after fitting the model on every virion, the estimated *V*_r_ and the geometry become clear and valid (b) and by relating reduced volumes to contact geometry, we gain information on adhesional compliances (Figure 5c) and size estimations.

**Figure 6:**
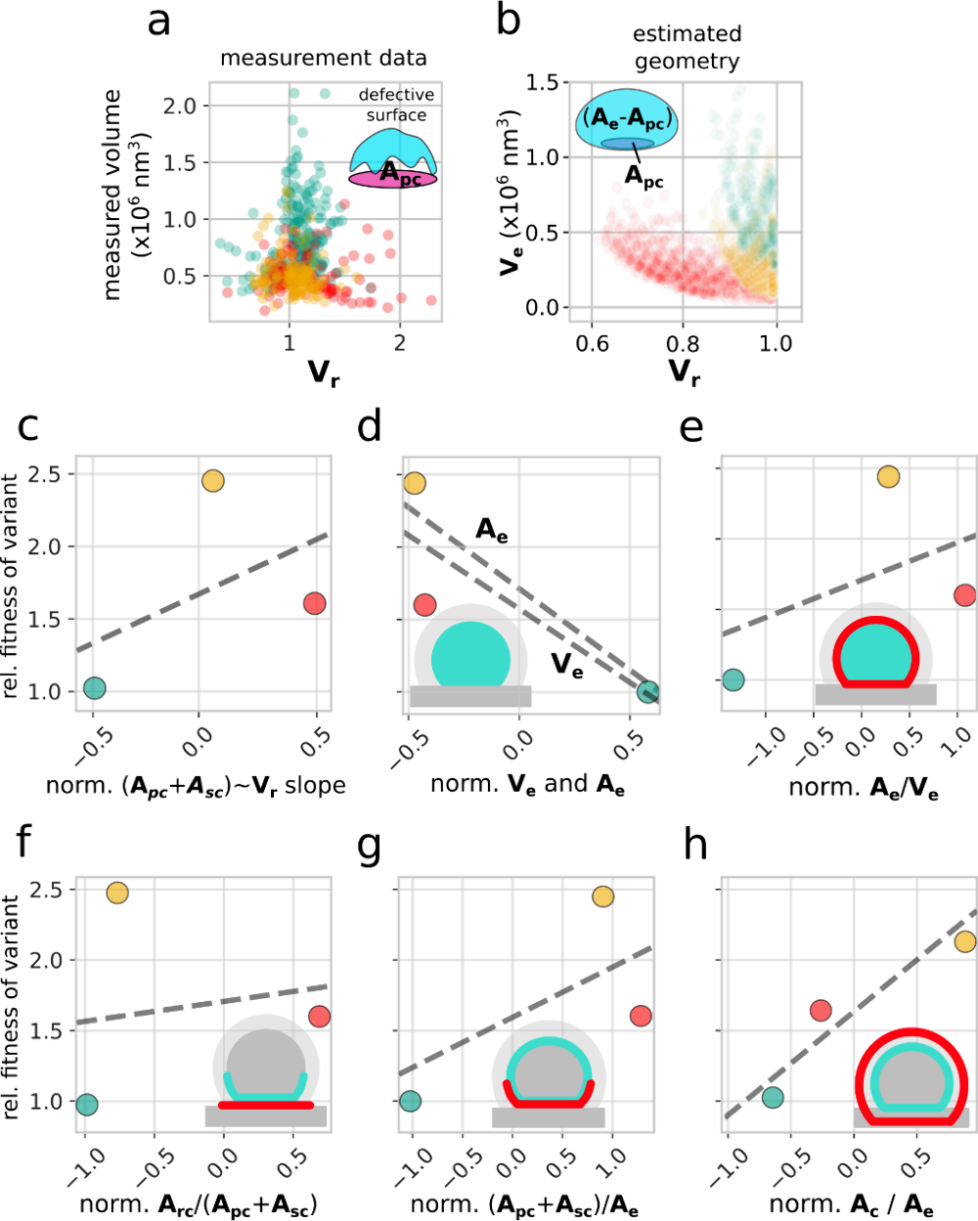
Overview of the study and numerical results. The definition of reduced volume is explained in detail in Text S4 and Equation S6. (a) Illustration of how unreliable the original reduced volume values are since the measured surface and projected areas are too noisy and can even miss surfaces (due to mask cutoff) on the sides of the virions (inset), *n* = 230 *wild-type*, 130 *delta*, 124 *alpha*. (b) After fitting the model on the topographies, the geometrical data become meaningful, *n* = 3228 *wild-type*, 2327 *delta*, 2520 *alpha*. (c–h) Indicative correlations of important estimated parameters with biological relative fitness.^52^ The variables are normalized for easier comparison. These OLS lines are descriptive trend summaries fit to variant-level-means (*n* = 3), so the curves should be interpreted as exploratory associations, not precise predictors (color coding is teal = *wild*, red = *alpha*, orange = *delta*).

To put our findings into the wider context of viral infectivity and transmissibility, we regressed the relative virus fitness values with the means of some of our estimated geometrical parameters (Figure 6c–h). With only three variants, the key information is the qualitative association; the absolute slope or correlation size is unstable and should not be overinterpreted. Our findings suggest that the observed virion features may be related to the biological fitness of the variants. The reduced volume vs contact area’s slope (Figure 6c) suggests that increased adhesional compliance (greater slope, on Figure 5c, right this means a less negative slope) may represent a biologically relevant feature, potentially related to subtle nanomechanical differences. The absolute volume and surface area measurements (Figure 6d) suggest a possible inverse association with relative fitness, raising the question of whether smaller overall virion size might contribute to variant-specific biological properties. Conversely, our defined geometrical ratios show a varying positive relationship with viral fitness (Figure 6e–h). It has been shown^10^ that the spike protein vibrational mechanics correlate with variant infectivity, and our study extends these observations to the whole virion level above the effects of the spike protein. Dynamic descriptors such as residence time, interface stability, or restriction of spike conformational freedom may prove useful in future AFM-based neutralization studies, but such quantities are not measured in the present work.

It is also worth noting that our study provides a new aspect on the featurization of virus–host contact geometry. This could have practical implications for relevant machine-learning models and systems that are trained to predict viral infectivity.

### Limitations of the Study

Our study used only topographical AFM images of the early SARS-CoV-2 variants. Due to the low number of variants included, our correlation results (Figure 6c–h) should be interpreted as indicative, and the small sample size does not allow the use of extensive, multiple linear models.

We first positioned the virions on the antibody-covered substrate surface and then applied glutaraldehyde (GA) as a fixative in an attempt to conserve the structure (Figure 3d). Generally, the effects of GA include the stiffening of the specimen,^32^ sample aggregation,^38^ and altering protein conformation.^31^ Particle aggregation was not a problem as we discarded aggregates from the analysis. Since we used only morphology to observe features, sample stiffening did not affect our analysis directly. Conformational alterations of individual proteins might introduce global morphological artifacts or change spike protein conformations, as demonstrated earlier.^23^ As the morphology can be affected by GA, the observed shapes on an absolute scale are somewhat uncertain; however, the vesicle sizes are almost identical with EM literature values (see the Model Fitting Reveals Further Differences between Variants section). This reinforces our modeling approach and shape estimations. Moreover, relative comparisons remain informative; as all samples and conditions in our study were processed identically, GA introduces a common offset.

The spike layer is the first protruding assembly that mediates contact with the surrounding environment and likely contributes to both receptor engagement and the emergent mechanical response of the virion. Recent AFM/HS-AFM studies have shown variant-dependent ACE2-interface mechanostability and antibody-bound mechanical behavior at the spike level,^40,59^ while isolated spike trimers also display rapid conformational fluctuations and hinge-mediated flexibility. The present work does not resolve such molecular mechanics directly; rather, it captures a whole-virion phenotype in which spike properties are one contributor among envelope proteins, lipids, and RNP organization. We also note that the substrate was functionalized with antispike antibody rather than ACE2; the model therefore assumes an effective uniformly accessible contact shell and does not attempt to resolve nanoscale ligand heterogeneity.

We model the adhered virion envelopes as Helfrich equilibrium vesicle shapes and retain only those solutions that satisfy the measured height and lateral size tolerances, i.e., equilibria under experimental geometric constraints. Both the Helfrich model and the adhesion phenomena possess several parameters. Our shape estimation model has only two topographical features (height and radius) as input, thus underconstraining the model; this makes only shape estimation possible to some extent without a straightforward extraction of specific nanomechanical moduli. Our contact metrics are physics-regularized through the fitted shapes, and the estimates are reported with uncertainty and statistical evaluation (Figures S2–S3, Tables S2–S3). These are more useful to reveal comparative trends rather than absolute values; also, our shape estimations have indirect support from the agreement of fitted envelope sizes/areas with literature ranges.

The particle counts were slightly unbalanced (230 *wild-type*, 130 *delta*, 124 *alpha*) reflecting a practical constraint on specimen availability and imaging throughput, not post hoc selection. We retained all quality-controlled particles to avoid the loss of precision and potential selection bias from discarding valid observations.

A clean way to isolate how variant-dependent nanomechanics and geometry affect infectivity would be to engineer isogenic virions that share an identical spike while varying only nonspike, variant-specific proteins. This enables a *ceteris paribus* comparison that removes spike-specific effects as confounders. Alternatively, with some limitations, like in ref 11, one can utilize virus-like particles for which exact parameter control is possible. To rule out fixation bias in any of the parameters, all variants should be scanned in both fixed and unfixed states as well. Given the biosafety challenges of highly infectious strains, however, large-scale computational simulations may be especially valuable as demonstrated also by ref 11.

## Conclusion

Our analysis of the AFM data was strengthened by a tunable modeling pipeline that makes simple topographical scans more accurate and informative and also handles several geometric uncertainties. Our measurements provide strong evidence that coronavirus variants have a marked difference in their geometrical appearance and adhesional compliance, possibly through subtle nanomechanical variations. This would reaffirm the diversity of the evolutionary landscape of variants in geometrical and biophysical dimensions. The apparent virion geometry supports the hypothesis that the *alpha* and *delta* variants may exhibit a more-compact morphology. Together with the observed differences in apparent adhesional compliance, reduced virion size may represent a biologically relevant variant-specific feature; however, whether these features contribute to any competitive or functional advantage remains speculative. The estimated geometrical properties can be used to feature virion contact geometries. Because our descriptors are derived from fixed particles and an underconstrained geometric model, they should be interpreted as comparative assay-specific phenotypes rather than direct predictors of infectivity or intrinsic nanomechanical constants. These results reveal important patterns in the broader context of structural and nanomechanical properties of enveloped coronavirus virions, uncovering new aspects and inspiring further study of the complex interplay among viral structural components.

## Methods/Experimental Section

### Virus Production

All virus preparation steps were performed under biosafety level 3 (BSL-3) conditions at the National Biosafety Laboratory, National Center for Public Health and Pharmacy, Budapest, Hungary. SARS-CoV-2 variants used in this study were: *wild-type* (Wuhan-Hu-1), *alpha-variant* (B.1.1.7), and *delta-variant* (B.1.617.2) obtained from the Hungarian National Collection of Highly Pathogenic Viruses. The variants were isolated from oropharyngeal swabs of confirmed COVID-19 patients identified by RT-PCR. For virus production, we used monolayers of Vero E6 (European Collection of Authenticated Cell Culture, Salisbury, U.K.) cell cultures with approximately 75% confluence, cultured in DMEM (Sigma-Aldrich) supplemented with 5% FBS (fetal bovine serum; EuroClone, Pero, Italy) and 1× Cell Culture Guard (PanReac AppliChem, Darmstadt, Germany) in a 5% CO_2_ environment. The primary viral isolate was sequenced and propagated two times in cell cultures containing VP-SFM serum-free, ultralow protein medium (Gibco, ThermoFisher Scientific) supplemented with l-glutamine (Sigma-Aldrich, Merck, Darmstadt, Germany). Four days after inoculation, the virus-containing medium was collected and centrifuged (3000*g*) to remove cellular debris. To concentrate the virus, the supernatant was ultracentrifuged (84,000*g*, 1.5 h, 4 °C) in 13.5 mL lockable plastic tubes using a Sorvall MTX-150 ultracentrifuge. The pellet was resuspended in 100 μL of VP-SFM.

### Substrate Surface Preparation

To stabilize the coronavirus particles on a smooth and flat surface, we used antispike antibody-functionalized mica. These surfaces were prepared as described previously^23^ (Figure 3, inset 1). Briefly, 100 μL of 10 μg/mL recombinant protein G was dropped onto the glutaraldehyde covered poly-l-lysine surface (Merck, Darmstadt, Germany) and incubated for 30 min. The surface was then washed carefully five times with 100 μL of phosphate-buffered saline (PBS). Then, 100 μL of 10 μg/mL SARS-CoV-2 Spike Glycoprotein Antibody (#abx376478, Abbexa Ltd., Cambridge, UK) was dropped on the surface followed by 1 h of incubation. Lastly, the surface was washed with 100 μL of PBS five times to remove the unbound antibodies. The antibody-coated surfaces were stored under PBS until application for up to 5 days at 4 °C.

The antibody used in this study was purchased before 2021 and was originally raised against an early SARS-CoV-2 spike (with only *wild-type* circulating). We therefore cannot assume identical capture affinity or tethering geometry across later variants; accordingly, all contact-related metrics are interpreted as comparative descriptors under a common assay surface rather than direct measures of native host cell binding.

### Preparation of SARS-CoV-2 Samples

An aliquot of approximately 20 μL of purified SARS-CoV-2 sample was pipetted onto the preprepared antispike antibody-coated substrate surface and incubated at 37 °C for 25–30 min. The process was repeated twice. Subsequently, we rinsed the surface with PBS to remove unbound virions. All the sample-loading and washing steps were carried out in a laminar-flow hood in BSL-3 conditions (at the National Biosafety Laboratory, National Center for Public Health and Pharmacy, Hungary). For AFM imaging of chemically fixed SARS-CoV-2, 100 μL 5% of GA solution (in PBS) was added, and the sample was incubated for approximately 1.5 h, ensuring both fixation and virus inactivation. Following these steps, the sample was carried to the AFM laboratory (Department of Biophysics and Radiation Biology, Semmelweis University) and loaded into the environmental scanner unit of the Cypher ES AFM instrument.

### AFM Imaging

An Asylum Research Cypher ES instrument (Oxford Instruments, Santa Barbara, CA) was used for AFM imaging. All measurements were performed at room temperature (25 °C). We used dynamic, amplitude modulated noncontact mode (AC) scanning utilizing BL-AC40TS (Olympus Corporation, Japan) cantilevers. The samples were under liquid, covered with ∼100 μL of PBS solution. The cantilever was oscillated near its resonance frequency (∼20 kHz) by using photothermal or piezo excitation. The low cantilever spring constant (∼0.09 N/m), imaging mode, and fixed sample ensured that the topography of the specimen was not distorted (Figure S8). Surface bound, individual viral particles were identified visually and scanned with high resolution.

Scanning areas ranged from 500 × 500 nm to 3 × 3 μm with a pixel size of ∼2 × 2 nm, a scan rate of ∼0.6 Hz, a 70% amplitude set point, and a free amplitude of ∼10 nm. Each coronavirus variant was measured from 2 to 4 independently prepared samples (see Virus Production) acquired on different dates. Each independently prepared sample was deposited on one or two substrate surfaces (see Substrate Surface Preparation) for AFM imaging. One or two BL-AC40TS cantilevers were used per sample. All measurements were performed by the same two operators. The number of usable images per prepared substrate surface was approximately 5–10, each containing 3–10 unique coronavirus particles.

### Image Processing and Data Collection

Image postprocessing and analysis were conducted semiautomatically using IgorPro (Wavemetrics, Lake Oswego, OR, USA) and Gwyddion.^60^ The process is explained step-by-step in Figure 1, Figure S1b and Figure S9. Briefly, after noise, line artifact, and planar deviation removal, deconvolution was performed using Gwyddion’s built-in algorithm,^61^ incorporating the known shape of a BL-AC40 cantilever tip for precise corrections (tip shape was reconstructed with blind estimation using EMS 80130-NB, AFM SPM NioProbe Calibration surface). Then, particles were selected via Otsu thresholding, which separates the background (flat surface) from the foreground (virions) of the topographical images consistently without any arbitrary parameters required. The resulting particle masks were manually refined, if needed. Manual mask refinement was restricted to obvious segmentation failures, such as merged particles, scan-edge artifacts, or debris-adjacent masks, and was not used to tune particle size after particle acceptance. The final data set consisted of 230 particles for the wild-type, 130 for the delta variant, and 124 for the alpha variant.

We extracted the height of each particle from the center of the mask and the average particle radius as presented in Figure 1b. Furthermore, we collected raw AFM measurement information, such as volume and surface area. We also calculated the circularity (isoperimetric quotient), which is a shape descriptor. This helps us quantify how close the shape of the mask of the given particle is to a perfect circle. It is calculated as the ratio of the area of the mask to the area of a circle having the same perimeter as the mask.^62^

### Biological Fitness Data Collection

Any trait of the virus that affects the growth, reproduction, immune evasion, and generation time is culminated in what we call viral fitness. Fitness is a quantitative representation of reproductive success. Fitness can be expressed as a relative quantity; in the case of SARS-CoV-2, the relative fitness of a given variant is relative to the fitness of the original, *wild-type* variant (relative lineage growth per viral generation (∼5.5 days)). In our study, we used data from ref 52 in which the authors modeled the fitness of different variant mutations using genomic databases with known spatiotemporal prevalence. Their implementation was a hierarchical Bayesian multinomial logistic regression model, and this model yielded robust and credible relative fitness values in concordance with previous observations.

### Statistical Analysis, Model Implementation, Numerical Solutions and Visualizations

Statistical analyses were carried out using Excel (Microsoft), Prism,^63^ and Python. Outliers were identified at the particle level using Prism’s ROUT method (*Q* = 1%) applied only to the raw AFM measurements used for model fitting, namely, particle height and mean radius, prior to shape generation. If either raw parameter of a given particle was flagged as an outlier, the entire particle was excluded from subsequent analyses. This procedure removed 8, 4, and 5 particles from the wild-type, alpha, and delta data sets, respectively. No additional outlier filtering was applied to the ensembles of generated shapes; all shapes derived from the retained particles were kept. For inferential comparison of the three groups, Welch’s one-way ANOVA (*scipy.stats.f_oneway*) was performed on virion-level mean values, followed by Games–Howell (*statsmodel.stats.multicomp.pairwise_tukeyhsd*) post hoc pairwise comparisons (see Figures S13, S14, S15 and Tables S1, S2, S3). Correlation coefficients and regressions were calculated with *scipy.stats.linregress* (see Table 2 and Table S4).

Differential equation solutions, other numerical calculations, and related data handling were implemented in Python using the Numpy,^64^ Scipy,^65^ and Pandas^66^ packages. For visualizations, we used the Matplotlib^67^ and Seaborn^68^ packages.

## Supplementary Material



## Data Availability

Interactive envelope visualizer (https://sirarthur100.github.io/coronavirus_variants/)

## Notes

Vesicle model implementation and additional statistics (PDF)
