# Supplementary material for: Unveiling the Structural and Mechanical Diversity of SARS-CoV‑2 Variants Using Atomic Force Microscopy
Source: ACS Appl Mater Interfaces. 2026 Jun 1;18(23):32325–38. doi: 10.1021/acsami.6c03486 (PMC13288390; doi:10.1021/acsami.6c03486)
Supplement: Supplementary file 1 [file am6c03486_si_001.pdf]

SUPPORTING INFORMATION

Unveiling the Structural and Mechanical Diversity of SARS-CoV-2 Variants Using Atomic Force Microscopy

Dominik Sziklai[1,], Bálint Budavári[1], Bálint Kiss[1,2], Levente Herényi[1], Zoltán Kis[3], Bernadett Pályi[3] and Miklós Kellermayer[1,2,*]*

[1] Department of Biophysics and Radiation Biology, Semmelweis University, Budapest, Pest county, 1094, Hungary

[2] HUN-REN-SE Biophysical Virology Research Group, Semmelweis University, Budapest, Pest county, 1085, Hungary

[3] National Biosafety Laboratory, National Center for Public Health and Pharmacy, Budapest, Pest county, 1097, Hungary

*To whom correspondence should be addressed at:

Dominik Sziklai: sziklaidominik09@gmail.com

Miklós Kellermayer: kellermayer.miklos@semmelweis.hu

Contents

**Text S1:**	*Vesicle shape theory*	*3*
**Text S2:**	*Corona layer*	*7*
**Text S3:**	*Shape modeling of the virion*	*8*
**Text S4:**	*Reduced volume*	*19*
**Figure S1:**	*Applied vesicle model and sample measurement data.*	*3*
**Figure S2:**	*Fitting pipeline and sensitivity/uncertainty analysis.*	*9*
**Figure S3:**	*Converting measured features to estimated envelope features through the fitting pipeline.*	*11*
**Figure S4:**	*Increasing grid search resolution minimizes numerical sampling artefacts.*	*12*
**Figure S5:**	*Estimated virion features as a function of grid resolution.*	*13*
**Figure S6:**	*Example envelope shape estimations for each variant.*	*14*
**Figure S7:**	*High-level architecture of the shape fitting algorithm.*	*15*
**Figure S8:**	*AFM amplitude induced virion deformation.*	*16*
**Figure S9:**	*Image processing workflow in Gwyddion.*	*17*
**Figure S10:**	A_{pc} *and* A_{rc}-A_{pc} *area comparisons of different variants.*	*20*
**Figure S11:**	*Receptor contact area (*A_{rc}*) increase is beneficial.*	*21*
**Figure S12:**	*Can shape reflect surface contact properties directly?*	*22*
**Figure S13:**	*Boxplots of measured features.*	*23*
**Figure S14:**	*Boxplots of modeled features.*	*24*
**Figure S15:**	*Boxplots of modeled ratios.*	*26*
**Table S1:**	*Statistical analysis of measured features.*	*23*
**Table S2:**	*Statistical analysis of modeled features.*	*24*
**Table S3:**	*Statistical analysis of modeled ratios.*	*26*
**Table S4:**	*Statistical analysis of correlation and regressions.*	*27*
**Equation S1:**	*Helfrich free energy*	3
**Equation S2:**	*Extension with Lagrange multipliers*	4
**Equation S3:**	*Second order ODE system*	5
**Equation S4:**	*First order ODE system*	5
**Equation S5:**	*Shape integration*	5
**Equation S6:**	*Reduced volume*	19

Text S1: *Vesicle shape theory*

The model is based on the Helfrich membrane bending energy and extends it to adhered vesicles. It assumes the F free energy functional of the vesicle in the form

$$F = -WA_{adh} + \oint \frac{1}{2}\kappa(c_1 + c_2)^2 dA\,, \tag{S1}$$

where c_1 and c_2 are the two local principal curvatures [m^{-1}] of the membrane (**Figure S1a**), κ [J] denotes the Helfrich bending constant, A_{adh} [m^2] is the contact area between the vesicle and the substrate, and W [Jm^{-2}] is the contact (adhesion) potential. dA denotes the area integral over the vesicle surface that is not in contact with the substrate.

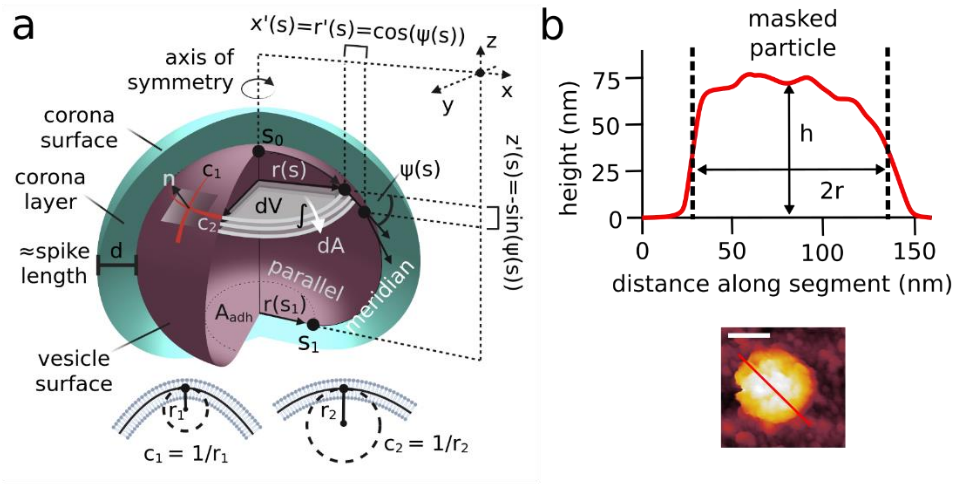

Figure S1. *Applied vesicle model and sample measurement data.* ***(a)*** *Visual explanation of the vesicle equilibrium model parameterization in 3D space (along the x, y and z axes). dV and dA are the infinitesimal volume and area elements respectively, which are integrated along the parameterized curve. At the bottom, we show the two principal curvatures in 2 dimensions. (See every parameter definition in* ***Text S1 and S2****)* ***(b)*** *Topographical plot profile (top) from an AFM image (below). The dashed lines indicate where the mask cuts off the particle (scale bar 50 nm).*

To control the area and volume of the vesicle, we extend the above functional with two Lagrangian multipliers[1], thus putting constraints on the resulting solutions of the variational problem:

$$F^* = F + \sigma A + PV \ , \qquad \text{(S2)}$$

where σ [Jm^{-2}] is the multiplier for the area constraint, P [Jm^{-3}] is the multiplier for the volume constraint, A [m^2] is the vesicle area and V [m^3] is the volume of the vesicle. The functional F^* in **SEq.2** can be realized by using axisymmetric contours parameterized by the arc length s of the meridians (also called profile curves). We define $\Psi(s)$ as the tilt angle of the meridians (**Figure S1a**). An auxiliary variable $r(s)$ is also introduced to measure the distance to the axis of revolution and ease the calculations. Visualized in **Figure S1a**, the following relationships are set: $x'(s) = r'(s) = cos(\Psi(s))$, $z'(s) = -sin(\Psi(s))$, $c_1 = \Psi'(s)$ and $c_2 = sin(\Psi(s))/r(s)$. The principal directions of c_1 and c_2 are the meridians and parallels of the surface of revolution.

After taking the first variation of F^* (**SEq.2**), the solution of the two emerging Euler-Lagrange equations yields a system of second-order differential equations[1]. An auxiliary Lagrangian field $\gamma(s)$ is also introduced to enforce the proper geometry (for details, see[1]). This system provides the minimum energy (equilibrium) vesicle solutions with the applied constraints. To solve this second-order differential equation system (**SEq.3**) numerically, it is rewritten into a system of 4 first-order equations (**SEq.4**).

From

$$
\begin{aligned}
0 &= \ddot{\psi} - \frac{\gamma \sin\psi}{\kappa r} - \frac{Pr\cos\psi}{2\kappa} + \frac{\dot{\psi}\cos\psi}{r} - \frac{\sin 2\psi}{2r^2} \\
0 &= \dot{\gamma} - \frac{1}{2}\kappa(\dot{\psi}^2 - \frac{\sin^2\psi}{r^2}) - \Sigma - Pr\sin 2\psi \\
\dot{r} &= \cos\psi
\end{aligned} \tag{S3}
$$

to

$$
\begin{aligned}
tmp &= \dot{\psi} \\
0 &= \dot{tmp} - \frac{\gamma \sin\psi}{\kappa r} - \frac{Pr\cos\psi}{2\kappa} + \frac{tmp \cdot \cos\psi}{r} - \frac{\sin 2\psi}{2r^2} \\
0 &= \dot{\gamma} - \frac{1}{2}\kappa(tmp^2 - \frac{\sin^2\psi}{r^2}) - \Sigma - Pr\sin 2\psi \\
\dot{r} &= \cos\psi
\end{aligned} \tag{S4}
$$

where the *tmp* variable was introduced to trade off one order. The initial condition is $r(s_0) \approx 0$, $\gamma(s_0) = 0$, $\Psi(s_0) = 0$[1-3] and $\Psi'(s_0)=tmp$ was varied as described later. The solution starts from s_0 until $\Psi(s)$ crosses π; this is where we reach the substrate (**Figure S1a**). After solving the system, we can render the curve (parameterized by s) back to the real shape of the vesicle in Cartesian coordinates by numerically integrating the vector valued function ($x'(s)$ and $z'(s)$ are indicated in **Figure S1a**) as

$$
\sum_{s_0}^{s_1} \begin{bmatrix} x'(s) \\ z'(s) \end{bmatrix} \Delta s = \sum_{s_0}^{s_1} \begin{bmatrix} \cos(\Psi(s)) \\ -\sin(\Psi(s)) \end{bmatrix} \Delta s\,. \tag{S5}
$$

After obtaining one meridian (profile curve) of the vesicle surface, we generate the surface by revolution around the z-axis to render the vesicle in 3 dimensions. Thus, the vesicle becomes a closed surface (with the addition of A_{adh}).

To summarize, this model provides a way of numerically fitting physically realistic vesicle shapes on our measurement data by using the obtained height and radius of the viral particles

(**Figure S1b**). Further information on possible approaches is provided in[4]. Our model treats vesicle membrane as an infinitesimal layer, no assumptions are made about its internal structure, so no elastic energy is attributed to tilt or splay separately[5]. Notably, the implemented vesicle equilibrium model was primarily developed for the description of giant vesicles, with lower curvatures compared to virions. Nevertheless, the basic idea presented here could be successfully adapted using more involved mathematical and physical considerations (e.g., including higher order curvature terms in the energy functional).

Text S2: *Corona layer*

Since the spikes are constrained to the vesicle membrane and point outwards, we can define a corona layer around the fitted vesicle, in which the spikes are present (**Figure S1a**). This corona layer (or shell) is bounded by the vesicle and the corona surface, which is constructed by translating each point x on the vesicle surface along its outward normal $n(x)$ with a constant distance d. Thus, each point x on the vesicle corresponds to a point x^* on the corona surface, where $x^* = x + d \cdot n(x)$. Given that the vesicle is convex and the scaling is performed outward, the created corona surface is also closed and convex, and it and encompasses the vesicle entirely.

Text S3: *Shape modeling of the virion*

To generate the equilibrium shape model, we need to solve a system of first-order differential equations (**SEq.4**) [1, 3]. To fit this model onto the specific virion height and radius values, we need to solve it as a boundary value problem, so that our vesicle model produces a vesicle with the same height and radius as the measured virion, that is $h_{model} \sim h_{measured}$ and $r_{model} \sim r_{measured}$. This fitting was implemented as a shooting method[6], with the mentioned -20 nm tolerance (main text, **Model Fitting Reveals Further Differences Between Variants section**). The shooting method briefly is changing the initial values of the ODE to acquire a solution for which the boundary conditions ($h_{model} \sim h_{measured}$ and $r_{model} \sim r_{measured}$) are satisfied. The shooting did not implement the classical root-finding step; we simply explored the parameter space (grid search) and collected the parameter combinations which provided a solution within tolerance limits. When we are interested only in geometry, we do not need to address which parameters of the model we are changing; we simply require that it produces a shape within the error boundaries.

The -20 nm tolerance was selected because of the observed spike length of virions on EM images[7] and 3D molecular reconstructions[8]. Because spike proteins on the virion surface introduce uncertainty in measured topographic heights, using their approximate physical length as a proxy for height variability imposes physically credible bounds on an otherwise purely numerical algorithm.

We defined the width of the corona layer as $d = 20\ nm$[4, 9], thus creating a shell around the modeled vesicle (**Figures S1a**). After the construction of the envelope shape, this corona layer allows us to extract several geometrical parameters in connection with virus-host interaction. Comparing virions along these geometrical parameters can give valuable insights and reveal differences.

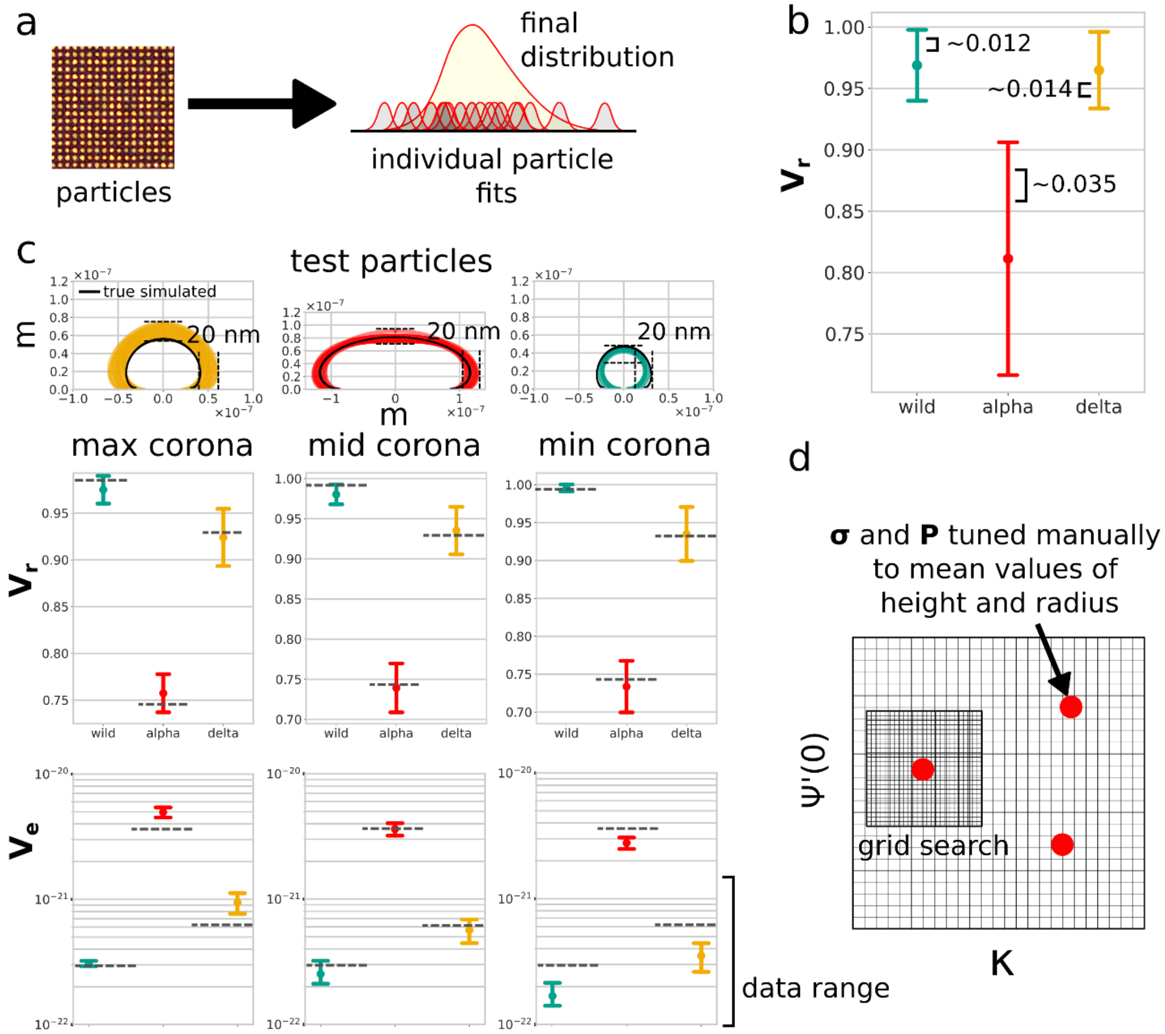

Figure S2. *Fitting pipeline and sensitivity/uncertainty analysis.* ***(a)*** *Several particles of the same kind are fitted, producing 15-20 fits on average. These shape fits represent the given variant.* ***(b)*** *V_r is a central shape parameter in our study, so we present its uncertainty in the fitted shapes. The means and standard deviations are shown for all the fits for the given variant, next to them in black, the average, per fit standard deviations are shown.* ***(c)*** *Simulated vesicles to assess how sensitive and uncertain the prediction is for a known shape. 3 types of vesicles, with substantially different shapes and sizes were tested. The top row illustrates how the 20 nm tolerance level distributes the fits. The first column illustrates the scenario, where the measured height and radius*

are extended by spikes with maximal error (20 nm). The middle column illustrates the case when the added error is 10 nm, and the last column shows the case when we only measure the vesicle without spikes. These are of course edge cases, real measurements are possibly a combinations of these. Nevertheless, we expect that the top and radial spike lengths should be between 0-20 nm, and in the middle, 10 nm, our fitting works best as can be seen in the middle column, the true values fall almost at the middle of our estimate. Both uncertainties for V_r and V_e estimations are shown. Most data in our study falls in the indicated data range. ***(d)*** *Schematic illustration of the fitting. Computationally, it is feasible that we first set the σ and P Lagrange multipliers manually, then sweep the other two parameters with grid search. The grids is varied with a small random value to reduce numerical artifacts during fitting.*

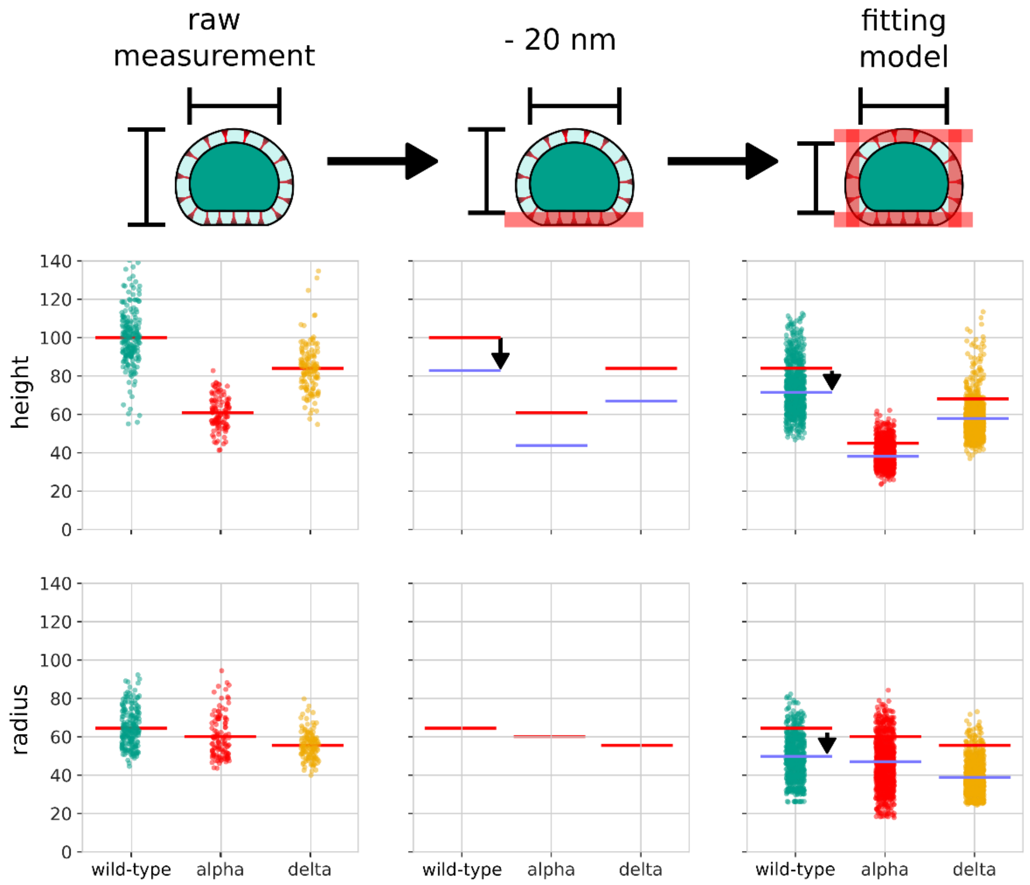

Figure S3. *Converting measured features to estimated envelope features through the fitting pipeline. Visualizing how the height and radius values change.*

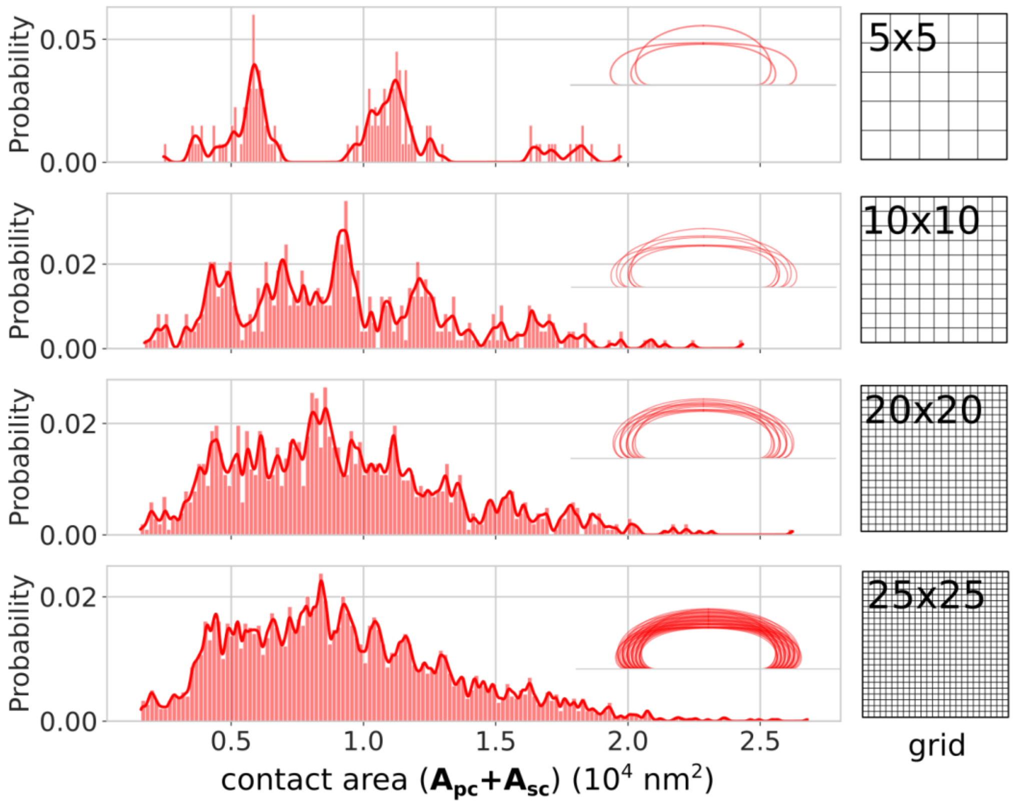

Figure S4. *Increasing grid search resolution minimizes numerical sampling artefacts. The contact area distributions (for alpha) are shown for the respective grid resolutions (grid values were produced on a log scale, equally distributed, using numpy's logspace function). A kernel density estimation is also overlayed on the histograms, along with representative fit ensemble for a variant envelope. As one can see, low grid (top) produce very few shapes for a given virion. Finer grids sample all possible shapes satisfying the tolerance threshold, so at around grid ~ 25x25, numerical sampling artefacts are minimized. Our final results used this 25x25 grid resolution.*

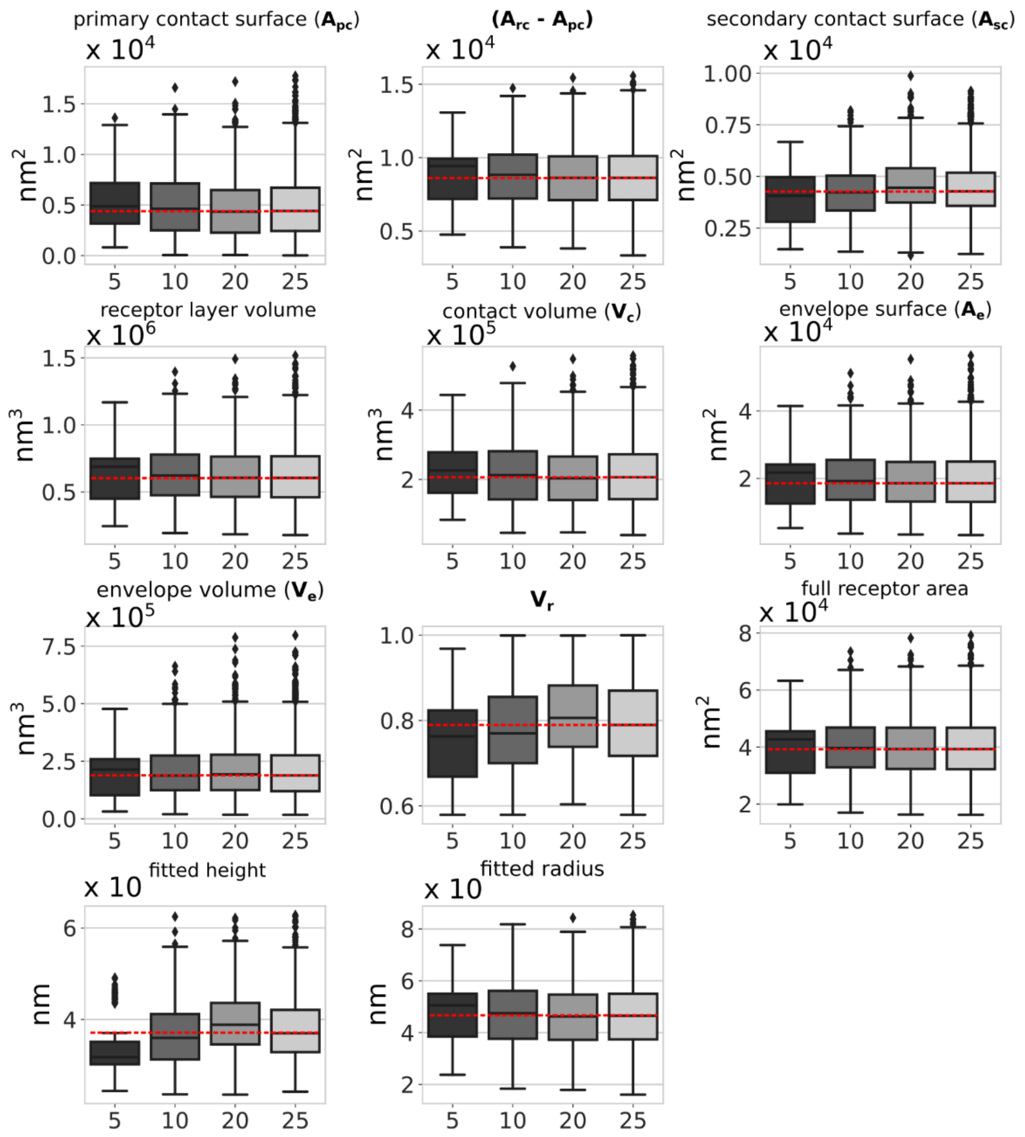

Figure S5. *Estimated virion features as a function of grid resolution. It can be seen that for finer grids, the estimated values are stabilized as the sampling becomes more exhaustive.*

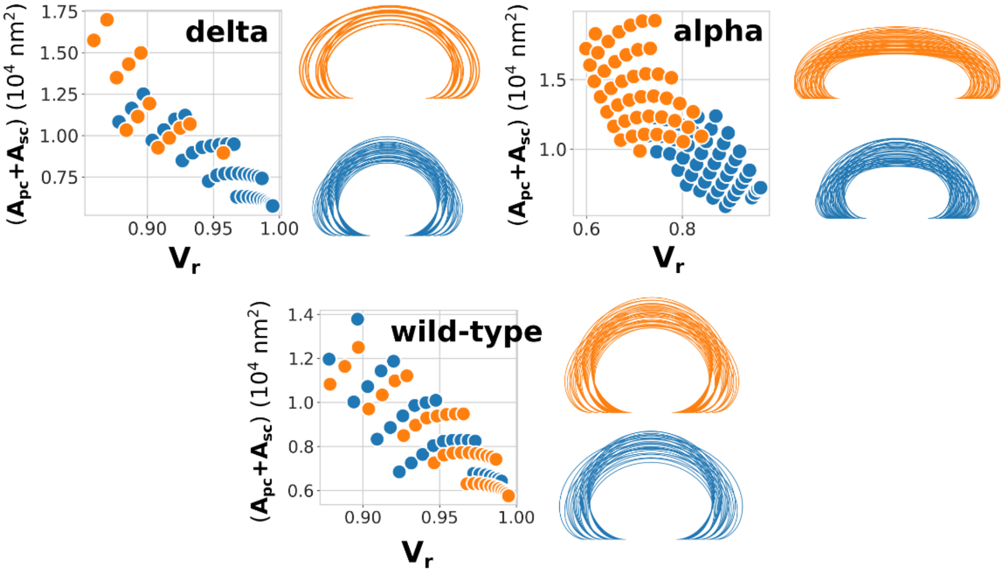

Figure S6. *Example envelope shape estimations for each variant.*

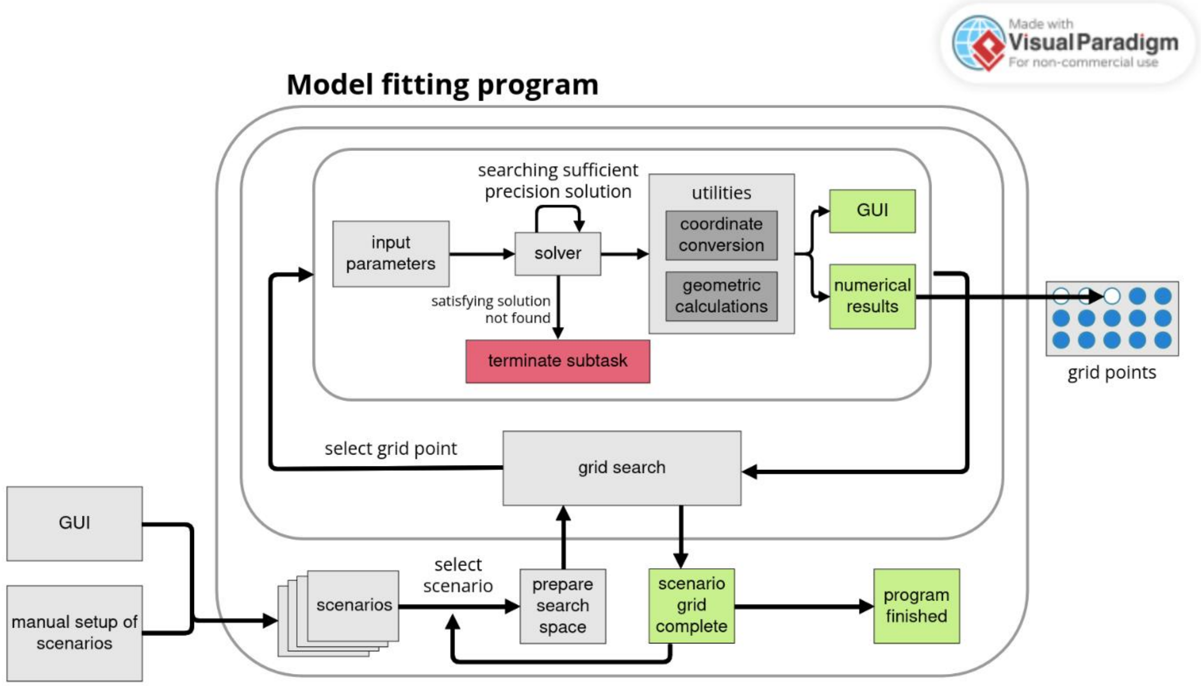

Figure S7. *High-level architecture of the shape fitting algorithm. The scenarios (initial parameters) are set up manually, then the grid search runs in an automatic fashion. When solving the differential equation system (**solver**), we expect the solution vector to contain at least a few thousand points within the region of interest, thus we can achieve a sufficient shape accuracy. This is achieved by tuning the step size of the Runge-Kutta solver. The independent nature of the grid elements allows one a fast implementation by utilizing parallel computations.*

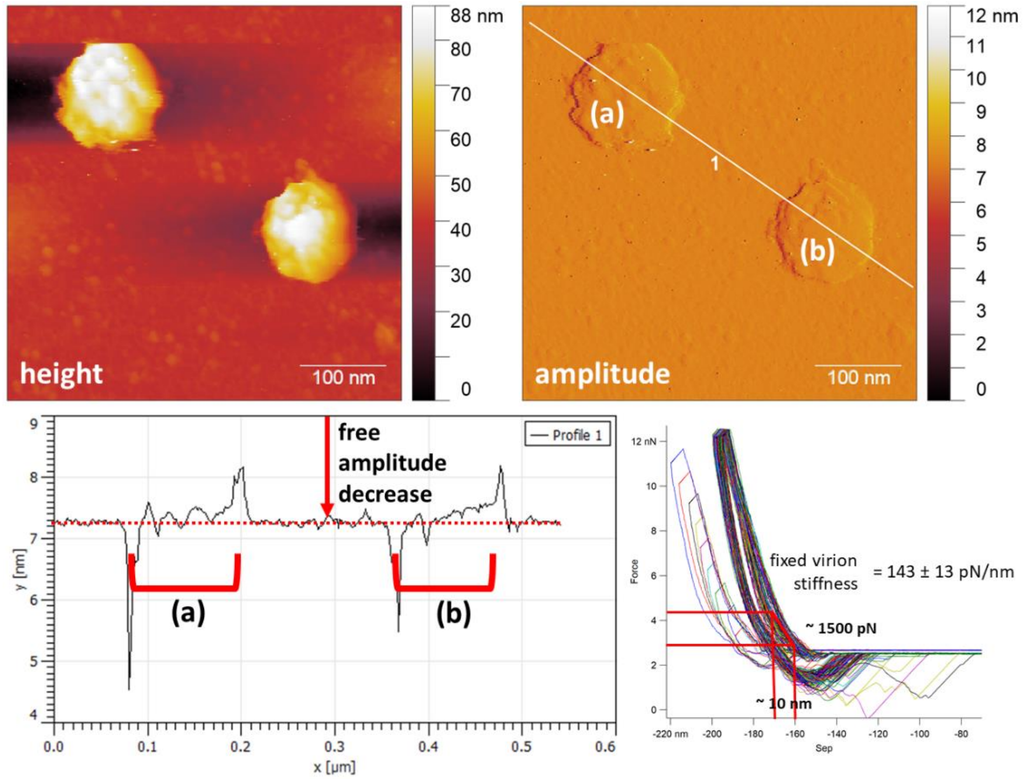

Figure S8. AFM amplitude induced virion deformation. A representative AFM topographical scan of two virions (**top left**). On the **top right**, we see the amplitude values during the scan as a result of feedback. **(Bottom left)** We see the extracted amplitude values along the segment, with the respective virions. With a 70% amplitude setpoint, and ~ 10 nm free amplitude. If one makes the very rough, quasi-static assumption that the amplitude reduction corresponds directly to cantilever deflection against the surface, the force scale would be $k*(10\text{-}7) \sim 270\,pN$. However, AM tapping does not provide a direct measure of peak force. The stiffness of unfixed coronavirus/influenza varies greatly (~13-50 pN/nm), and as our samples are fixed, this value is expected to be substantially higher. Indeed, our indentations of fixed virions provided stiffness value estimates, **bottom right**, ~ 143 pN/nm. Comparing this to the 270 pN/nm value exerted by the tip, we deduce that morphological deformation due to tapping is minimal (~ 2 nm)

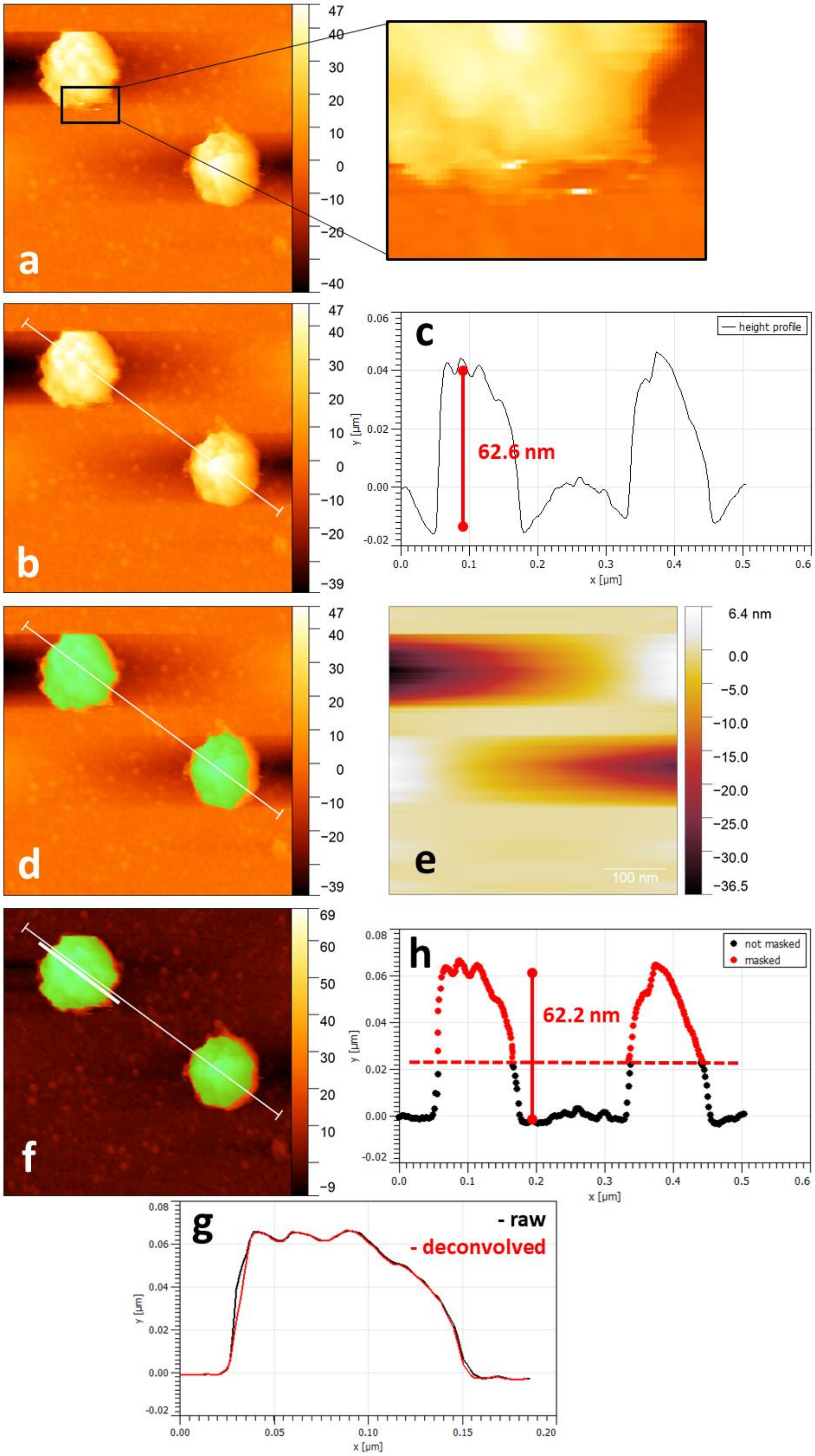
a
b
c
d
e
f
g
h
height profile
62.6 nm
62.2 nm
not masked
masked
- raw
- deconvolved
6.4 nm
100 nm
x [µm]
y [µm]

Figure S9. *Image processing workflow in Gwyddion.* ***(a)*** *First the scars and outliers are removed from the topographical scans.* ***(b)*** *The denoised topographical scan and the corresponding height along the segment* ***(c)****.* ***(d)*** *Usage of Otsu's method to distinguish foreground from background, thus selecting the virions. Horizontal row alignment with* 2nd *order polynomial* ***(e)*** *to remove scan line artefacts.* ***(f)*** *The corrected topography, on which we utilize the deconvolution.* ***(g)*** *The result of deconvolution provided only very slight morphological changes as indicated.* ***(h)*** *After the recalculation of masks using Otsu's method* ***(f)*** *on the processed images, we acquire the final height segment, showing the masked (red) and unmasked (black) section. This also shows us that the mask cutoff is approximately at the FWHM (Full Width at Half Maximum).*

Text S4. Reduced volume

To consistently reference the shapes and flatness of generated shape, we use their dimensionless reduced volume[1], which is defined as

$$V_{rv} = \frac{V}{\frac{4}{3}\pi(\frac{A}{4\pi})^{\frac{3}{2}}}, \tag{S6}$$

where V is the volume and A is the surface area of the vesicle, so that the smaller the V_r, the more deflated the vesicle.

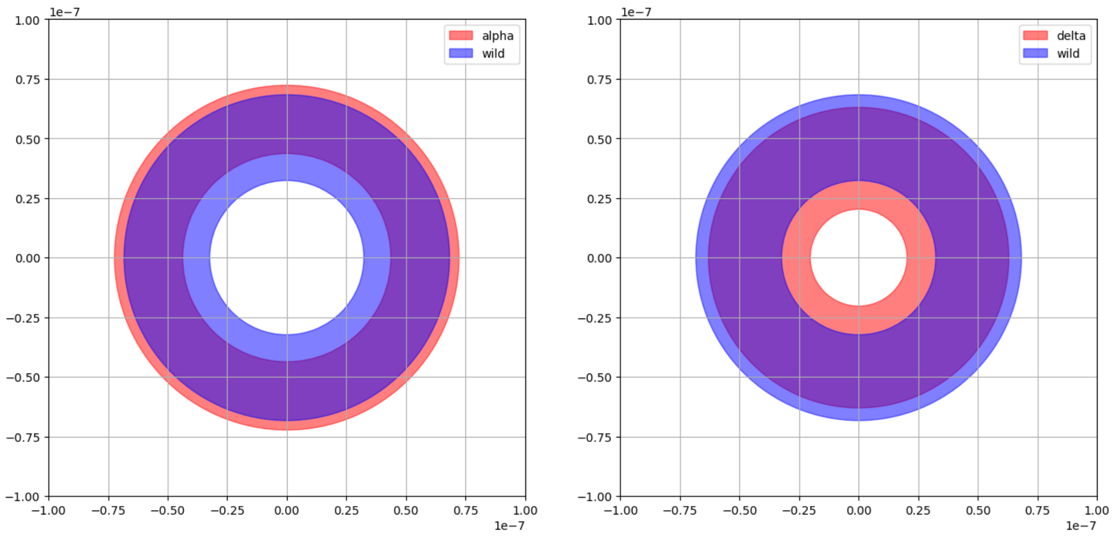

Figure S10. A_{pc} and A_{rc}-A_{pc} area comparisons of different variants.

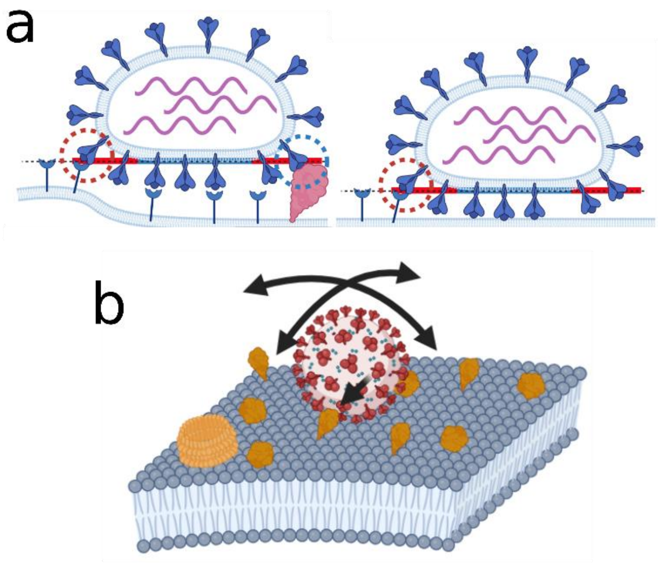

Figure S11. *Receptor contact area (A_{rc}) increase is beneficial, as we can clearly see on* ***(a)*** *two specific scenarios where the extra area can engage in extra interaction, either specific or non-specific.* ***(b)*** *illustrates how this extra area facilitate viral exploration of surfaces.*

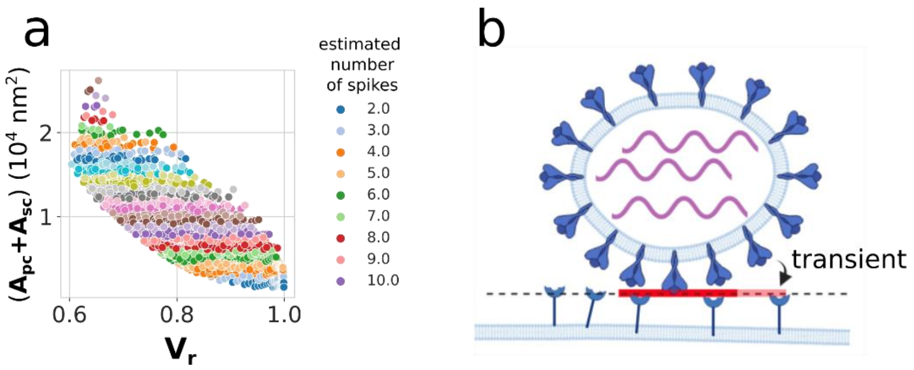

Figure S12. *Can shape reflect surface contact properties directly?* ***(a)*** *Using previously published estimates of spike densities*[7]*, we can estimate the expected number of spikes at the contact zone assuming an equal spike distribution over the envelope. One could even expect that the engaged spike proteins could influence what contact area states are mostly occupied for a given variant, as we presume that transient states* ***(b)*** *between spike-surface engagements are less preferred (from a contact area point-of-view). This would imply that changes of contact area statistically happen in a somewhat staircase like manner, synchronously with spike-surface interactions. Unfortunately, our data - with relatively high uncertainty - is not suitable for such analysis to detect the abundance of such peaks on the contact area histogram (e.g.* ***Figure S4****).*

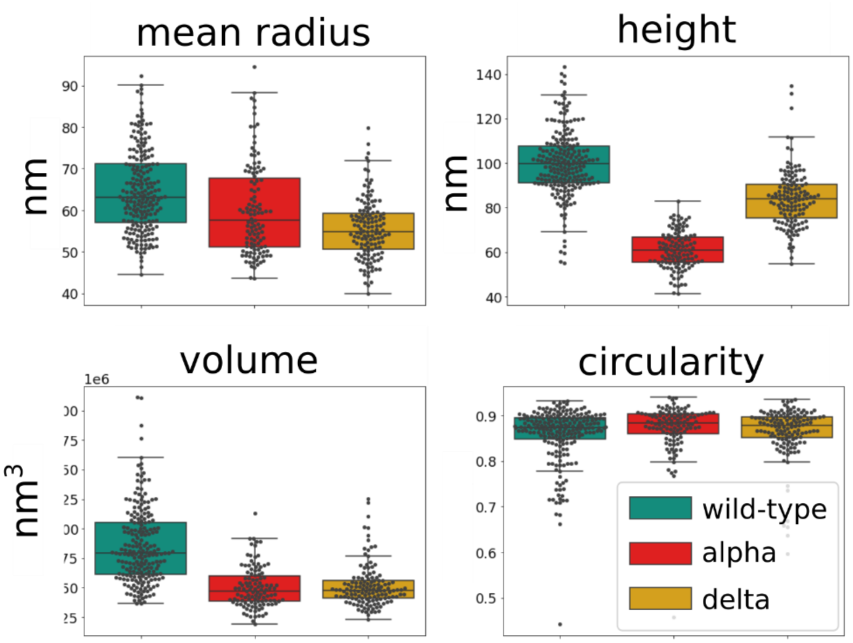

Figure S13. *Boxplots of measured features. n* = 230 *wild-type*, 130 *delta*, 124 *alpha.*

Measured feature	***ANOVA (p)***	**Games–Howel *(p & Cohen's d)***		
		alpha vs wild-type	***alpha vs delta***	***wild-type vs delta***
mean radius	*	*<br>*0.43*	*<br>*0.49*	**<br>*1.00*
height	***	***<br>*3.01*	***<br>*2.10*	**<br>*1.13*
volume	**	***<br>*1.27*	***ns***<br>*0.07*	**<br>*1.24*
circularity	***ns***	-	-	-

Table S1: Statistical analysis of measured features. * ($p < 0.05$), ** ($p < 0.01$), *** ($p < 0.001$), *n* = 230 *wild-type*, 130 *delta*, 124 *alpha.*

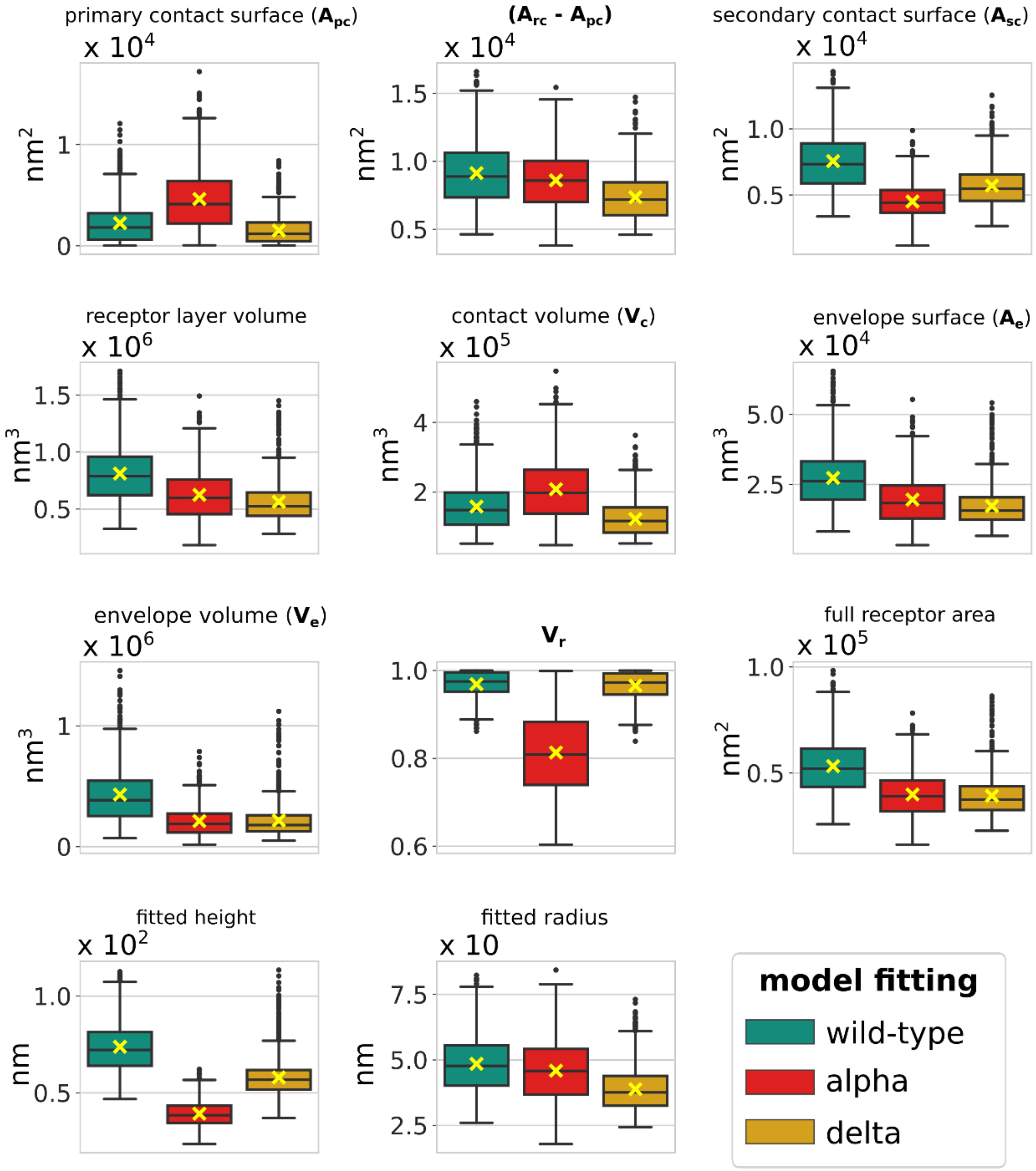

Figure S14. *Boxplots of modeled features. n =* 3228 *wild-type*, 2327 *delta*, 2520 *alpha.*

Modeled feature	*ANOVA (p)*	**Games–Howel *(p & Cohen's d)***		
		alpha vs wild-type	*alpha vs delta*	*wild-type vs delta*
primary contact surface (A_{pc})	**	**<br>*0.88*	**<br>*1.32*	*<br>*0.47*
(A_{rc}-A_{pc})	*	*<br>*0.24*	*<br>*0.64*	**<br>*0.88*
secondary contact surface (A_{sc})	**	**<br>*1.74*	**<br>*-0.82*	**<br>*1.01*

receptor layer volume	**	**<br>0.77	*<br>0.30	***<br>1.14
contact volume (V_c)	***	*<br>0.59	**<br>1.17	**<br>0.59
envelope surface (A_e)	**	**<br>0.79	*<br>0.28	**<br>1.14
envelope volume (V_e)	***	***<br>1.18	ns<br>0.03	***<br>1.12
reduced volume	***	***<br>2.01	***<br>2.17	ns<br>0.11
receptor contact surface (A_{rc})	***	***<br>1.09	ns<br>0.06	**<br>1.2
height	***	***<br>3.56	***<br>2.29	**<br>1.42
radius	**	*<br>0.22	**<br>0.66	**<br>1.02

Table S2: Statistical analysis of modeled features. * ($p < 0.05$), ** ($p < 0.01$), *** ($p < 0.001$), n = 230 *wild-type*, 130 *delta*, 124 *alpha.* For inferential comparisons of fitted shape parameters, each virion was treated as the independent experimental unit. For each parameter, all fitted shapes belonging to a given virion were summarized by their mean, and statistical comparisons were then performed on these virion-level mean values. Accordingly, the sample size for hypothesis testing was the number of virions, not the number of fitted shapes.

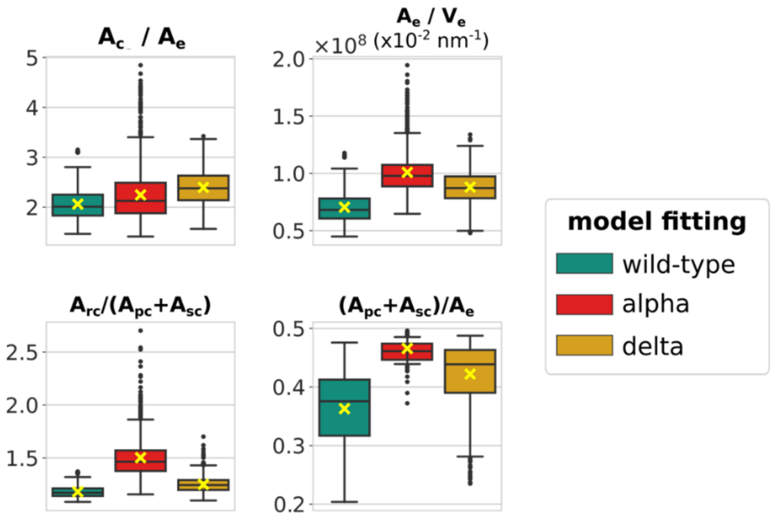

Figure S15. *Boxplots of modeled ratios. n* = 3228 *wild-type*, 2327 *delta*, 2520 *alpha.*

Modeled ratios	***ANOVA (p)***	**Games–Howel *(p & Cohen's d)***		
		alpha vs wild-type	***alpha vs delta***	***wild-type vs delta***
A_c/A_e	*	* *0.41*	* *0.34*	** *0.99*
A_e/V_e	**	*** *1.83*	** *0.78*	*** *1.23*
$A_{rc}/(A_{pc}+A_{sc})$	***	*** *2.18*	*** *1.78*	** *1.04*
$(A_{pc}+A_{sc})/A_e$	***	*** *2.57*	** *1.22*	*** *1.08*

Table S3: Statistical analysis of modeled ratios. * ($p < 0.05$), ** ($p < 0.01$), *** ($p < 0.001$), *n* = 230 *wild-type*, 130 *delta*, 124 *alpha.* For inferential comparisons of fitted shape parameters, each virion was treated as the independent experimental unit. For each parameter, all fitted shapes belonging to a given virion were summarized by their mean, and statistical comparisons were then performed on these virion-level mean values. Accordingly, the sample size for hypothesis testing was the number of virions, not the number of fitted shapes.

	wild	alpha	delta
**measured correlations (95% CI)**	n = 230 *wild-type*, 130 *delta*, 124 *alpha*		
average rad. vs height	0.12 (-0.01 – 0.26) **ns**	0.09 (-0.09 – 0.27) **ns**	0.31 (0.15 – 0.46) *
height vs vol.	0.42 (0.30 – 0.53) *	0.70 (0.61 – 0.79) **	0.71 (0.61 – 0.79) **
average rad. vs vol.	0.90 (0.87 – 0.92) ***	0.65 (0.53 – 0.74) **	0.82 (0.76 – 0.87) ***
**measured slopes (±SE)**	n = 230 *wild-type*, 130 *delta*, 124 *alpha*		
average rad. vs height	0.19 ±0.1	0.07 ± 0.07	0.55 ± 0.14
height vs vol. (nm^3 x nm^{-1})	9138 ± 1364	14634 ± 1345	9942 ± 854
average rad. vs vol. (nm^3 x nm^{-1})	29264 ± 1008	10184 ± 1119	20266 ± 1231
**estimated correlations (95% CI)**	n = 230 *wild-type*, 130 *delta*, 124 *alpha*		
V_r vs (A_{pc}+A_{sc})	-0.85 (-0.87 - -0.83) **	-0.77 (-0.79 - -0.76) *	-0.77 (-0.79 - -0.76) **
V_r vs fitted height	0.07 (0.00 – 0.15) **ns**	0.46 (0.43 – 0.49) **	0.18 (0.15 – 0.22) *

Table S4: Statistical analysis of regressions. * ($p < 0.05$), ** ($p < 0.01$), *** ($p < 0.001$), For inferential comparisons of fitted shape parameters, each virion was treated as the independent experimental unit. For each parameter, all fitted shapes belonging to a given virion were summarized by their mean, and statistical comparisons were then performed on these virion-level mean values. Accordingly, the sample size for hypothesis testing was the number of virions, not the number of fitted shapes.

References

1. Tordeux, C.; Fournier, J.; Galatola, P., Analytical characterization of adhering vesicles. *Phys Rev E* **2002,** *65*, 041912.

2. Seifert, U.; Lipowsky, R., Adhesion of vesicles. *Phys. Rev. A.* **1990,** *42* (8), 4768-4771.

3. Seifert, U.; Berndl, K.; Lipowsky, R., Shape transformations of vesicles: Phase diagram for spontaneous- curvature and bilayer-coupling models. *Phys. Rev. A.* **1991,** *44* (2), 1182-1202.

4. García-Arribas, A. B.; Ibáñez-Freire, P.; Carlero, D.; Palacios-Alonso, P.; Cantero-Reviejo, M.; Ares, P.; López-Polín, G.; Yan, H.; Wang, Y.; Sarkar, S.; Chhowalla, M.; Oksanen, H. M.; Martín-Benito, J.; de Pablo, P. J.; Delgado-Buscalioni, R., Broad Adaptability of Coronavirus Adhesion Revealed from the Complementary Surface Affinity of Membrane and Spikes. *Adv Sci* **2024,** *11*, 2404186.

5. Kozlov, M. M.; Taraska, J. W., Generation of nanoscopic membrane curvature for membrane trafficking. *Nat Rev Mol Cell Biol* **2023,** *24* (1), 63-78.

6. Won Y. Yang, W. C., Jaekwon Kim, Kyung W. Park, Ho-Hyun Park, Jingon Joung, Jong-Suk Ro, Han L. Lee, Cheol-Ho Hong, Taeho Im, Applied Numerical Methods Using Matlab. Wiley: Hoboken, New Jersey, 2020; pp 305-374.

7. Ke, Z.; Peacock, T. P.; Brown, J. C.; Sheppard, C. M.; Croll, T. I.; Kotecha, A.; Goldhill, D. H.; Barclay, W. S.; Briggs, J. A. G., Virion morphology and on-virus spike protein structures of diverse SARS-CoV-2 variants. *The EMBO Journal* **2024,** *43* (24), 6469-6495.

8. Choi, Y. K.; Cao, Y.; Frank, M.; Woo, H.; Park, S. J.; Yeom, M. S.; Croll, T. I.; Seok, C.; Im, W., Structure, Dynamics, Receptor Binding, and Antibody Binding of the Fully Glycosylated Full-Length SARS-CoV-2 Spike Protein in a Viral Membrane. *J Chem Theory Comput* **2021,** *17* (4), 2479-2487.

9. Ke, Z.; Oton, J.; Qu, K.; Cortese, M.; Zila, V.; McKeane, L.; Nakane, T.; Zivanov, J.; Neufeldt, C. J.; Cerikan, B.; Lu, J. M.; Peukes, J.; Xiong, X.; Kräusslich, H.-G.; Scheres, S. H. W.; Bartenschlager, R.; Briggs, J. A. G., Structures and distributions of SARS-CoV-2 spike proteins on intact virions. *Nat.* **2020,** *588* (7838), 498-502.
